# Analysis of Flaring Activity at Liquefied Natural Gas (LNG) Export Facilities Worldwide

**DOI:** 10.1021/acs.est.5c03755

**Published:** 2025-09-20

**Authors:** Laura Minet, Forood Azargoshasbi, Meredith Franklin, Gunnar W Schade, Margaret J. McGregor, Kate McInnes, Tim K. Takaro

**Affiliations:** † Department of Civil Engineering, 8205 University of Victoria, Victoria, British Columbia V8P 5C2, Canada; ‡ Institute for Integrated Energy Systems, University of Victoria, Victoria, British Columbia V8P 5C2, Canada; § Department of Statistical Sciences and School of the Environment, 7938 University of Toronto, Toronto, Ontario M5S 1A4, Canada; ∥ Atmospheric Sciences, Texas A&M University, College Station, Texas 77843, United States; ⊥ Department of Family Practice, Faculty of Medicine, 8166 University of British Columbia, Vancouver, British Columbia V6T 1Z3, Canada; # Investigative Journalist & Researcher, Squamish, British Columbia V8B 0A1, Canada; ∇ Faculty of Health Sciences, Simon Fraser University, Burnaby, British Columbia V5A 1S6, Canada

**Keywords:** liquefied natural gas (LNG), flaring, LNG export facilities, environmental impact assessment, Visible Infrared Imaging Radiometer Suite (VIIRS), VIIRS Night Fire (VNF)

## Abstract

Liquefied natural gas (LNG) export facilities are booming worldwide to supply gas for the growing energy demand. Flaring, the controlled burning of natural gas, occurs at these facilities during operations ranging from start-up to ongoing maintenance and under emergency situations. Although flaring can be a significant air pollutant and greenhouse gas emission source, little information exists on the frequency, duration, and volume of gas flared by LNG export facilities. This study leveraged ten years of data from the Visible Infrared Imaging Radiometer Suite (VIIRS) Night Fire (VNF) product associated with 48 existing LNG export facilities globally to develop probabilities of flaring at different life-cycle stages. We found a significantly higher volume of gas flared in the first two years of a facility’s operation (i.e., on average 1.9 (1.0–3.2) billion cubic meters (bcm) per capacity vs 0.62 (0.43–0.92) bcm during subsequent years). During regular operations, the annual volume of gas flared was correlated with the facility’s production capacity, and flaring varied greatly among facilities (148 (137–159) flaring days/year on average and 0.73 (0.64–0.85) bcm/capacity). Unfortunately, most environmental assessments overlook the start-up phase and fail to consider worst-case scenarios. As flaring is a source of air pollution, its potential health impacts on local populations may be underestimated in these assessments.

## Graphical abstract

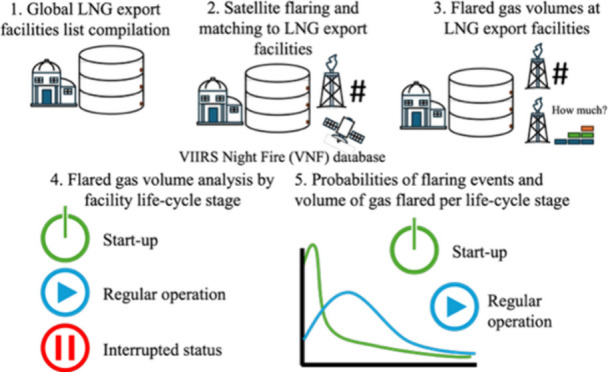

## 1 Introduction

The rapid global expansion of liquefied natural gas (LNG) export facilities has outpaced the availability of objective data on their actual flaring activities. This gap complicates estimates of real environmental impacts and may weaken regulatory oversight and public health protection.

Global energy demand is rising and natural gas, composed primarily of methane (CH_4_), but also hydrocarbons, nitrogen (N_2_), carbon dioxide (CO_2_), helium (He), hydrogen sulfide (H_2_S), and noble gases, depending on its source,^1^ has increasingly been used to satisfy this demand. By cooling gas to −162 °C, exporting countries convert it into LNG^2^ for efficient storage and shipment. Over the past decade, numerous LNG export facilities have opened, particularly in Australia and the U.S., with many more in planning stages there and in other countries including Canada, Mexico, and Qatar.^3–6^

Although LNG facilities are designed to maximize exports, certain stages require flaring, the controlled combustion of natural gas. Flaring mainly occurs during start-up (when testing and calibrating the facility), maintenance (when parts of the system are nonfunctional and the liquefaction process cannot be entirely completed), and shutdown events (to empty pipes), and is typically used as a safety measure when equipment becomes overpressurized.^7^

Flaring emits fine particulate matter (PM_2.5_), volatile organic compounds (VOCs), carbon monoxide (CO) and nitrogen oxides (NO_*x*_),^8–11^ all linked to adverse health outcomes.^12–15^ Although epidemiological research specifically examining the health effects of fossil gas flaring is limited,^16^ one study found that maternal exposure to a high number of nightly flare events during unconventional oil and gas extraction was associated with an increased risk of preterm birth.^17^

In most countries including the U.S.,^18^ Canada,^19^ and Australia,^20^ permits are required before construction of an LNG export (or import) facility, and operators are typically required to conduct an environmental impact assessment that includes an estimate of gas flaring frequency, duration and volume.^21–24^ However, many assessments are outdated, sometimes approved a decade before construction.^23,25^ Regulators generally rely on the operator’s estimates of flaring since there is no publicly available data on the volumes of natural gas typically flared by export facilities.^26^ These estimates rarely account for flaring during the facility’s start-up period, and assume limited maintenance and emergencies during regular operations.^23,27^ Reporting is inconsistent, and enforcement weak,^28^ leaving actual flaring poorly documented.

Satellite instruments capable of detecting thermal anomalies such as the Visible Infrared Imaging Radiometer Suite (VIIRS)^29^ capture flaring events, providing objective flaring information that can be linked to existing LNG export facilities. VIIRS data are publicly available, yet no comprehensive benchmark of global LNG export facility flaring exists. In this study we assessed the number of flaring events and amounts of natural gas flared at LNG export facilities using the VIIRS Night Fire (VNF) product,^30^ which provides data since 2012. We developed probabilities of occurrence of flaring and associated estimates of volumes of gas flared at LNG export facilities during different life-cycle stages to better inform future environmental impact assessments.

## 2 Methodology

Our methodology (Figure S1 in the Supporting Information—SI) includes: (1) compiling a list of global LNG export facilities from several sources; (2) matching VNF flare events with the identified facilities; (3) linking VNF and World Bank (WB) annual flaring estimates; (4) analyzing flaring volumes by life-cycle stage of the facilities; and (5) developing probabilities of flaring per life-cycle stage.

### 2.1 Global LNG Export Facility List Compilation

We extracted a list of global LNG facilities operating since the 1970s and/or still operating from the World Bank (WB) Global Gas Flaring Data Web site.^31^ Using the database Global Energy Monitor (GEM) wiki,^32^ we distinguished import from export facilities, retaining only the export facilities, and recording their capacity and start year. To complete the WB list, we added facilities listed as “LNG Export Facilities” in the Environmental Defense Fund (EDF) “Oil and Gas Infrastructure Mapping”^33^ database. Data on facilities’ export capacity and start year were again drawn from the GEM wiki database, and we compiled a final list from the WB and the EDF datasets.

### 2.2 Satellite Flaring and Matching to LNG Export Facilities

We used the VNF dataset,^34^ which identifies nightly flaring events since the launch of the Suomi National Polar-orbiting Partnership satellite in 2012.^35^ The VNF product provides information on flare location (latitude and longitude), temperature, radiative heat intensity, and the sky conditions (i.e., cloud mask) at the time of detection. To isolate flaring events specifically associated with LNG export facilities identified in Section 2.1, we applied two filters:

1) Spatial filter: we excluded flares occurring more than 750 m from an LNG export facility. This threshold aligns with the resolution of the VIIRS instrument (750 m).^36^ A sensitivity analysis using a 1 km radius produced the same results.
2) Temperature filter: We removed detections with temperatures below 1,100 K, following WB guidance for excluding nonflaring thermal sources (e.g., biomass burning).^37^ While some studies^30,36^ have used higher temperature thresholds for broader geographic analyses, our focus on high temperature events near known LNG export facility sites where no other thermal sources with such high temperatures exist justified the 1100 K cutoff. Cloud cover, which can interfere with detection of flares by absorbing radiant emissions^30^ and limiting the detection of small thermal sources with relatively low temperature,^38^ was not used as an exclusion criterion due to our focus on high-temperature events. However, we conducted a sensitivity analysis to assess potential impacts (see SI Section S2.2).

Due to the bowtie effect,^39^ which corresponds up to a 15% overlap between successive orbits near the equator,^40^ an LNG export facility can be observed twice by the VIIRS instrument on the same night. To avoid double counting, records were merged so only one flaring event per facility per day was kept. We also analyzed consecutive-day flaring streaks as an indicator of persistent flaring.

Finally, to avoid misattributing flaring events, we used the “Oil and Gas Infrastructure Mapping”^33^ database to identify any LNG export facilities located within 750 m of other oil or gas infrastructure. These facilities were excluded from the analysis to prevent flare misattribution.

### 2.3 Flared Gas Volume at LNG Export Facilities

Both the WB Global Gas Flaring Data^31^ and VNF datasets provide estimates of annual flared gas volume by location (i.e., latitude and longitude). These estimates are derived from radiative heat intensity emitted by the VIIRS-detected flares, calculated based on blackbody temperature and source area. The radiative output is then converted into volume of gas flared using an empirical relationship developed from country-level data compiled by Cedigaz,^41^ an international organization that aggregates gas flaring statistics from most countries worldwide.^37,42^

For the WB dataset, we aggregated multiple records per facility per year. For the VNF dataset, Keyhole Markup Language (KML) files containing annual flared gas volumes were used to merge records from the same facility based on spatial coordinates, which was needed only for a few facilities (e.g., Algeria LNG, where trains are reported separately but analyzed here as one unit). We then aggregated total annual flaring volume per facility for the period 2012–2022.

We compared WB and VNF datasets by looking at the total annual volume of gas flared at each facility, to assess consistency and identify potential discrepancies.

### 2.4 Flaring Volume Analysis by Facility Life-Cycle Stage

Three phases of operation of an LNG export facility were distinguished:

1) Commissioning and start-up phases: commissioning precedes the start-up, involving thousands of safety tests on power systems, pipelines, tanks, and safety equipment. Most natural gas introduced is disposed of by flaring.^43^ During the start-up phase, the functioning of the facility is fine-tuned and flaring typically occurs intermittently,^44^ ranging from minutes to hours over weeks.^45^
2) Regular operations: flaring occurs during maintenance, turnarounds, and emergencies (safety measures to release pressure and prevent explosions).^44^ Routine operations involve annual equipment replacements, requiring complete natural gas removal for worker safety.^46,47^ Postmaintenance safety tests also require flaring before restart.^41^
3) Irregular operations: nonroutine flaring, which differs from emergency and routine flaring,^48^ occurs when operations are interrupted or stopped for technical, regulatory, or economic reasons, requiring lines to be emptied.^49^ Restarting after shutdown also causes significant flaring.^48^

To understand how the number of flaring events and the volume of gas flared varies across the lifespan of LNG export facilities, we analyzed flaring frequency and volume at each facility under four cases, comparing facilities with respect to their processing capacity.

#### 2.4.1 Case 1: Start-Up Conditions

This case only includes facilities that started after 2012, when VIIRS started collecting data. As start-up length is undefined (usually anticipated to be >1 year^50^), we investigated the effect of the length of this period ranging from 1 to 4 years.

#### 2.4.2 Case 2: Regular Operating Conditions—No Start-Up

This case analyzes the impact of potential maintenance operations or emergency conditions on flaring. We considered data from all facilities after *N*_year_ of operation, with *N*_year_ defined as the start-up period identified in case 1.

#### 2.4.3 Case 3: Regular vs Irregular Conditions—Continuous Operations vs Interrupted Status

For facilities under regular operating conditions, this case considered all operating years regardless of when the facility started operating. We included all facilities that showed a continued operating status since 2012. Facilities with an interrupted status (i.e., irregular conditions) since 2012, identified as “Mothballed” or “Idling” in the GEM wiki database,^32^ were considered separately.

#### 2.4.4 Case 4: Offshore vs Onshore

This case compares offshore and onshore facilities to analyze whether those two types of facilities display differences in their flaring operations.

### 2.5 Probabilities of Flaring Events and Volumes of Gas Flared per Life-Cycle Stage

We assessed flaring behavior during the start-up and regular operation phases of a facility. Specifically, we independently estimated the expected values for the following metrics: (1) the number of flaring days per year; (2) the number of consecutive flaring days; and (3) the annual volume of gas flared per capacity of the facility. These statistics were modeled across a range of event probabilities (from 10 to 90%) (i.e., 1—exceedance probability) to capture variability in flaring intensity. We tested a range of distributions to represent these outcomes: log-normal, generalized extreme value (GEV), Gumbal, exponential, and generalized Patero. Based on a goodness of fit evaluation (Section S3.4.1), we applied the log-normal distribution to all three variables, as described in eq 1.

Let *Y*(*t*) represent the annual value of a flaring-related statistic in year *t*. The cumulative distribution function (CDF) of the log-normal distribution is defined as

$$F(y)=\{\begin{array}{ll} 0, & y\leq \xi \\ \Phi \left(\frac{\ln(y-\xi )-\mu }{\sigma }\right), & y>\xi \end{array}$$ 1

where ξ is the location parameter; μ and σ are the mean and standard deviation of the log-transformed data, respectively; Φ denotes the standard normal CDF.

We estimated the parameters μ, σ, and ξ using the probability weighted moments method, which has been shown to produce more reliable estimates than maximum likelihood estimation when the sample size is small.^51^

To assess goodness of fit of the log-normal model, we applied two standard statistical tests: the one-sample Kolmogorov–Smirnov test,^52^ which evaluates whether a sample is drawn from a given reference probability distribution, and the one-sample Cramér–von Mises test,^53^ which provides a more sensitive measure of overall distributional fit. These tests help confirm whether our model accurately represents the observed data.

## 3 Results

### 3.1 LNG Export Facilities around the World

We identified 61 LNG export facilities worldwide by combining the WB Global Gas Flaring Data database^31^ (36 facilities) and the “Oil and Gas Infrastructure Mapping”^33^ database (25 additional facilities). Three facility clusters were located within <750 m from each other, closer than the spatial resolution of VIIRS, and were treated as single entities in this study: (1) AP LNG, Gladstone and QC LNG (Australia); (2) MNLNG, DM LNG, SM LNG and TM LNG (Malaysia) and (3) Vysotsk LNG and PO LNG (Russia). For each group, flaring data and facility capacities were aggregated. While this approach may slightly overestimate the number of flaring days or consecutive flaring days, possibly introducing outliers, the metric of volume of gas flared per capacity remained robust when summed. Section S2.1 of the SI provides detailed information on each facility/group of facilities.

We excluded 13 facilities from the final analysis based on the following criteria:

- Proximity to other oil and gas infrastructure: Freeport LNG and Snohvit LNG are located within 750 m of oil and gas facilities, raising uncertainty in flare source attribution;
- No detected flaring activity: No flares were identified for Tilbury Island LNG, Satu FLNG, Snurrevarden LNG, Risavika LNG, Hialeah LNG, Fort Nelson LNG and DF LNG;
- Missing flare volume data in VNF: While some flaring activity was visually identified for Tjelbergodden LNG, Kollsnes LNG, and Ichthys LNG, no corresponding gas volume estimates were available in the VNF dataset;
- Extreme outlier: Kiyanly LNG exhibited unusually high flaring activity compared to others with an average of 51.3% volume of gas flared per capacity, far exceeding the maximum average among the other regularly operating facilities (3.7%, see Table S1). Including this outlier would have disproportionately influenced the results.

After exclusions, 48 LNG export facilities were retained for analysis (Table 1). Approximately half (22) were commissioned prior to 2012 (pre-VIIRS, no start-up flaring data). Since 2012, 20 new facilities opened, including 6 in the U.S. and 7 in Australia. Among the 48 facilities included in our study, 4 showed irregular operations since opening.

**Table 1:** Summary of the 48 LNG Export Facilities Included in Our Analysis Grouped by Country, with Average Flaring Data Provided for 2012–2022^a^

	number of facilities			number of facilities				
country	onshore	offshore	start year	operational	inactive	total capacity (mtpa)	average number of annual flaring days	average volume of gas flared per capacity (%—bcm/bcm)
Algeria	2	0	1981–2013	2	0	16.1–25.3	299 (290–307)	2.82 (2.39–3.33)
Angola	1	0	2012–2012	1	0	5.2–5.2	119 (99–145)	1.90 (1.08–3.19)
Argentina	1	0	2019–2019	0	1	0.0–0.45	112 (106–117)	4.58 (3.40–6.10)
Australia	7	2	2006–2019	9	0	24.9–80.5	121 (105–135)	0.97 (0.60–1.45)
Brunei Darussalam	1	0	1973–1973	1	0	7.2–7.2	119 (106–134)	0.80 (0.54–1.04)
Cameroon	0	1	2018–2018	1	0	0.0–2.4	25 (12–48)	0.41 (0.08–1.05)
Egypt	2	0	2005–2005	2	0	8.2–12.2	170 (131–214)	1.22 (0.91–1.58)
Equatorial Guinea	1	0	2007–2007	1	0	3.7–3.7	72 (56–91)	0.68 (0.56–0.81)
Indonesia	3	0	1998–2015	3	0	21.1–30.6	107 (97–116)	0.63 (0.43–0.81)
Libya	1	0	1970–1970	0	1	3.2–3.2	321 (308–330)	7.54 (6.54–8.64)
Malaysia	4	0	1983–1983	4	0	43.2–43.2	137 (122–154)	0.25 (0.17–0.34)
Mozambique	0	1	2022–2022	1	0	0.0–3.4	167 (167–167)	12.8 (12.8–12.8)
Nigeria	2	0	2008–2019	2	0	22.0–22.8	132 (114–151)	0.57 (0.47–0.66)
Oman	1	0	2006–2006	1	0	10.4–10.4	126 (107–146)	0.25 (0.22–0.28)
Papua New Guinea	1	0	2013–2013	1	0	0.0–8.3	58 (32–86)	0.42 (0.14–1.15)
Peru	1	0	2010–2010	1	0	4.45–4.45	21 (12–35)	0.08 (0.05–0.13)
Qatar	2	0	2010–2011	2	0	77.4–77.4	305 (289–319)	0.33 (0.27–0.40)
Russia	4	0	2009–2019	4	0	10.0–31.1	94 (71–118)	0.61 (0.37–0.99)
Trinidad and Tobago	1	0	2007–2007	1	0	12.0–12.0	220 (197–244)	0.76 (0.66–0.86)
U.S.	7	0	1969–2022	6	1	1.5–102	53 (33–70)	0.16 (0.09–0.27)
UAE	1	0	1994–1994	1	0	7.6–7.6	319 (307–329)	2.03 (1.86–2.20)
Yemen	1	0	2010–2010	0	1	6.7–7.2	223 (179–259)	0.73 (0.44–1.10)

a mtpa and bcm stand for million tonnes per annum and billion cubic meters, respectively. Numbers in brackets correspond to the 95% confidence interval (2.5–97.5%).

### 3.2 Flares Detected at Each LNG Export Facility

For each facility, Table S1 provides the average number of flaring days per year identified by VNF since 2012 or since opening if later. Annual details are provided in Table S5. Only two facilities showed fewer than 20 flaring days per year since opening: Kenai LNG (U.S.—mothballed), and Elba Island (U.S.—opened in 2019). Thirteen facilities averaged more than 100 flaring days per year over 2012–2022, with many more exceeding 100 flaring days in some years. Table S1 also provides the average number of consecutive days with flaring during the complete study period.

Occasionally, we observed flaring occurring at facilities before their opening date, likely reflecting pilot flares. We also observed more flaring days after 2016, but it is unclear whether this reflects improved detection or false positives, though we have no reason to suspect the latter.

### 3.3 Volumes of Gas Flared at Each Facility

We observed notable discrepancies between the WB Global Gas Flaring Data^31^ and VNF^34^ datasets in facility-level estimated flared gas volumes. For approximately two-thirds of the facilities, WB reported lower estimates than VNF. From 2012 to 2022, facility-level differences in total flared gas volume ranged from 0 to 0.7 billion cubic meters (bcm) per facility (Table S6). Figure 1 compares the number of flaring days per year and the annual volumes of gas flared per unit of capacity for selected facilities, using both datasets. Results for the remaining facilities are in Figure S4. We found several WB estimates that warranted further investigation. In particular, for Marsa LNG, NLNG, and Gorgon LNG, the VNF dataset indicated a high number of flaring days, yet the WB dataset reported near-zero volumes of flared gas (e.g., 2013–2020 at NLNG, and 2012–2021 at Marsa LNG). This discrepancy suggests possible WB underreporting at certain sites. Given these discrepancies and the VNF dataset’s ability to capture both frequency and intensity of flaring events, we elected to rely on VNF-derived estimates for gas flaring volumes in our analysis.

**Figure 1:**
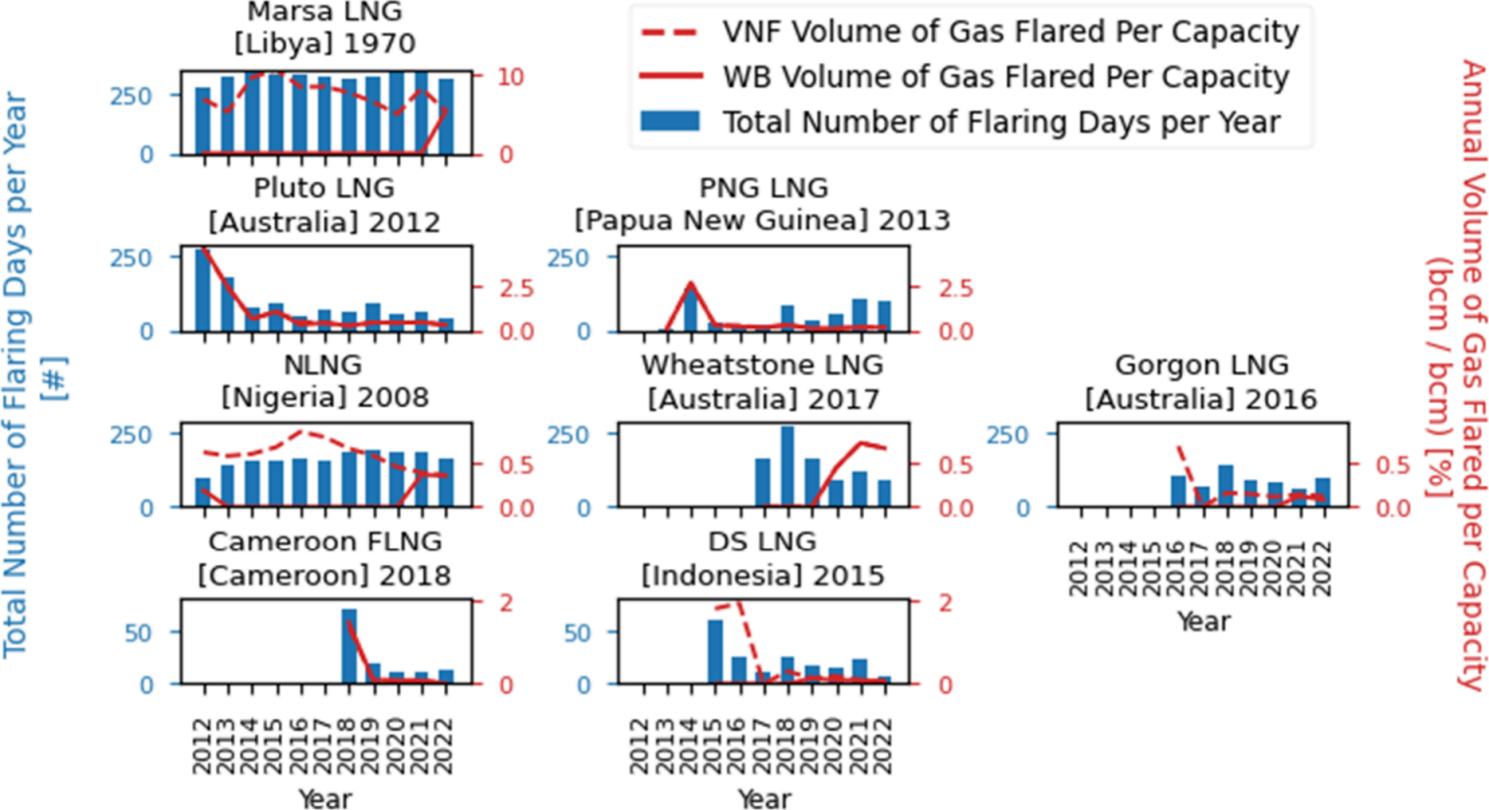
Annual number of flaring days per facility and comparison of the yearly volumes of gas flared per capacity provided by the World Bank (WB) and VIIRS Night Fire (VNF) datasets for a selection of facilities. When dashed lines corresponding to the VNF estimates do not appear, it is because they are hidden by the solid line of the WB estimates (in that case, VNF and WB estimates are the same). The name of each facility is followed by its country (in parentheses) and the year it opened. The facilities are ordered by flaring activity; scales may vary between graphs, but facilities with similar orders of magnitude are grouped together.

### 3.4 Analysis by Case

#### 3.4.1 Case 1: Start-Up Conditions

There were 22 post-2012 facilities with data for at least three operating years. After merging colocated sites AP LNG, Gladstone and QC LNG (Australia) and Vysotsk LNG and PO LN (Russia) due to their proximity (<750 m), 19 facilities remained for analysis.

##### 3.4.1.1 Volume of Gas Flared per Capacity

Figure 2 compares the distributions of annual volumes of gas flared per unit of facility stratified by duration of start-up (1–4 years). On average (median), facilities flared 2.4% (0.71%) of their capacity in year 1 vs 0.71% (0.28%) later, a marginally statistically significant difference (*p* < 0.1) (Table S10). However, when considering a two-year start-up period, average (median) flaring was 1.9% (0.70%) and 0.62% (0.25%) in later operational years (Table S10). This statistically significant difference (*p* < 0.05) represented a 3.2-fold increase in flaring volume per capacity. The 95% percentile of flaring during start-up was 7.2% compared to 2.3% in regular operational years.

**Figure 2:**
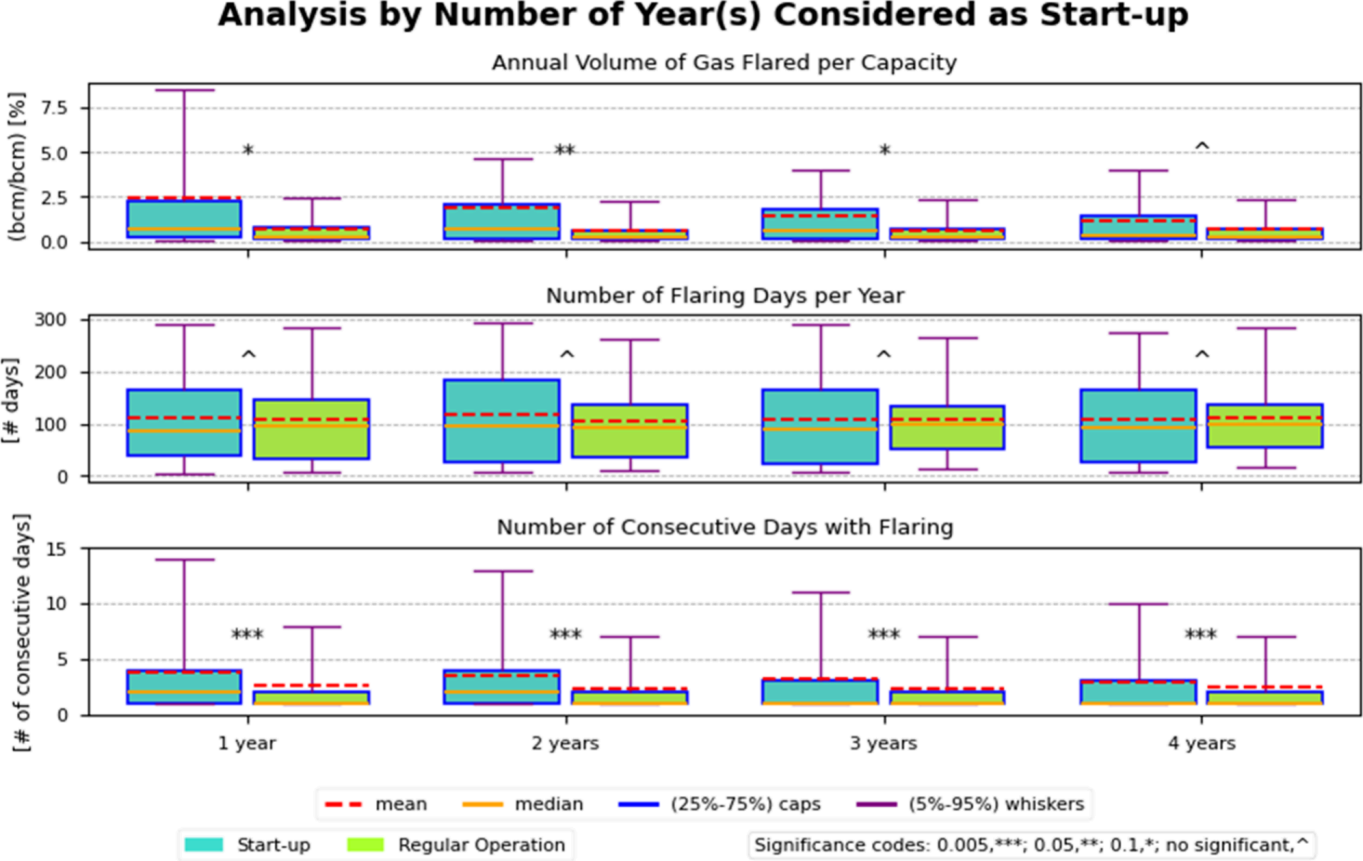
Analysis of the annual volume of gas flared per capacity, number of flaring days, and number of consecutive days with flaring by number of years considered as start-up for the 22 facilities that opened from 2012. The significance codes are based on *t* tests between start-up and regular operation conditions.

Facilities with largest start-up flaring per capacity included Angola LNG, DS LNG, Pluto, Prelude FLNG, and Yamal LNG (average/median 3.1%/2.2% in the first two years vs 0.90%/0.52% later; Section S3.1.3)

A 3 year start-up window showed a 2.1-fold higher average flaring during start-up compared to later years (*p* < 0.1), although medians were similar.

We found no significant Pearson or Spearman rank correlations between the amount of gas flared and the capacity of the facilities that started in 2012 and after. Additionally, comparisons between facilities commissioned before and after 2016 had no meaningful differences (Table S11).

##### 3.4.1.2 Number of Days with Flaring

During the first year of operation, facilities flared on average (median) 111 (86) days per year, compared to 108 (95) days during subsequent, regular operation years, a nonsignificant difference.

Differences were larger with a 2 year start-up window (Table S8), likely due to some facilities commencing operations late in the calendar year, resulting in continued start-up into the second year. That diminished with 3–4 years, but results remained statistically nonsignificant. Overall, the data suggest a higher flaring frequency during the first 2 years of operation.

##### 3.4.1.3 Number of Consecutive Days with Flaring

During a 1 year start-up, the average (median) number of consecutive flaring days was 3.8 (2.0) vs 2.5 (1.0) during subsequent regular operating years. For a 2 year start-up, the corresponding values were 3.6 (2.0) vs 2.4 (1.0) days. These differences were statistically significant (*p* < 0.001), indicating a higher persistence of flaring events early in operations.

#### 3.4.2 Case 2: Regular Operating Conditions—No Start-Up

To assess flaring during regular operation, we excluded the first two years of each facility’s operations, based on findings from Case 1, which showed significantly elevated flaring during this start-up period. Thus, we selected *N*_year_ = 2. For instance, Atlantic LNG, which opened in 2007, was considered out of start-up in 2009; therefore, we used all available post-2012 VIIRS data in the analysis. Similarly, Angola LNG which opened in 2012, contributed data starting from 2014. For newer facilities such as Cameron and Vysotsk LNG, which opened in 2019, only data for 2021 and 2022 were included in our analysis.

Volumes of gas flared per a facility’s capacity for these regular operational years are in Table S14; Figure S5 shows annual flaring days, consecutive flaring days, and flared gas volume per capacity for facilities operating under regular conditions.

A Spearman rank correlation coefficient of 0.51 (*p*-value <0.001) was observed between facility capacity and volume of gas flared among facilities with at least 3 years of operation. This contrasts sharply with the lack of correlation observed during start-up (Case 1), suggesting flaring during regular operations is more predictable and scales with facility size, while start-up flaring is more variable and harder to anticipate because less tied to capacity.

#### 3.4.3 Case 3: Regular vs Irregular Conditions

We aggregated data from all years for the 48 facilities with continuous operations since start-up and compared with the 4 facilities that had an interrupted status. Detailed results are in Section S3.2.

Facilities with interrupted operations typically experience shutdowns requiring flaring for purging pipelines or maintaining idling states. As a result, they exhibited substantially higher flaring activity, even when start-up years, typically associated with elevated flaring, were included.

From 2012 to 2022, the average (median) number of flaring days per year was 145 (127) for continuously operating facilities, and 215 (251) for interrupted facilities. This corresponds to an average (median) number of consecutive flaring days of 3.2 (1.0) for continuously operating facilities, and 4.8 (2.0) for interrupted operations. Annual volume of gas flared per capacity was 0.84 (0.47%) for continuous operations, and 3.6 (1.7%) for the interrupted facilities. These differences were statistically significant (*p* < 0.005).

When pooling data across all facilities and operational years without separating start-up, we again observed a Spearman rank correlation coefficient of 0.51 (*p* < 0.001) between capacity and annual volume of gas flared. This aligns with Case 2 and underscores that flaring during steady-state regular operation is related to facility size, unlike during start-up (Case 1), where no such correlation was found. As expected, no significant correlation between volumes of gas flared and capacity were observed for interrupted facilities, due to fluctuating flaring patterns.

#### 3.4.4 Case 4: Offshore vs Onshore

We compared onshore and offshore LNG export facilities for flaring activity (mean/median, Section S3.3). Flaring days per year were higher for onshore facilities (147/130) than offshore facilities (107/71). Consecutive flaring days were similar, 3.2 (2.0) for onshore and 3.2 (1.0) for offshore. Volume of gas flared by capacity was 0.75% (0.47%) for onshore and 2.0% (0.56%) for offshore facilities. Differences in flaring days and volume of gas flared per capacity were only marginally statistically significant (*p* < 0.1), suggesting offshore sites may flare less frequently but with greater intensity. These differences are unlikely due to detection over water vs land. VIIRS uses thermal infrared bands, which show no onshore–offshore bias. In fact, flare detection is certainly easier offshore due to the uniform background and lack of interferences.

### 3.5 Probabilities of Flaring Events and Associated Volumes of Gas Flared per Life-Cycle Stage

We derived probabilities for key flaring metrics across start-up (assuming 2-year start-ups) and regular operational phases. Using fitted log-normal distributions, we estimated expected values for the annual number of flaring days (computed with respect to all nights in the year), number of consecutive days with flaring, and yearly volumes of gas flared by capacity. Estimates were calculated for event probabilities of 10 and 90%. The values are presented in Table 2 and can be interpreted as “in a given year of the start-up (or regular operation) phase, there is *x*% probability a facility will flare *y*% of its capacity for *z* days or over n consecutive days”. Details of the log-normal distribution fitting and goodness of fit evaluation are presented in Section S3.4.2. These probabilistic insights provide a robust reference for future environmental impact assessments and regulatory planning, enabling a more realistic accounting of flaring across LNG facility life-cycle stages.

**Table 2:** Flaring Events and Associated Volumes of Gas Flared per Life-Cycle Stage (i.e., Start-Up and Regular Operating Years), Estimated Independently from Each Other, for Various Probabilities (1—Exceedance Probability)^a^

operation type	probability [%]	yearly volumes of gas flared per capacity (bcm/bcm [%])	number of days with flaring per year (days)	number of consecutive days with flaring (days)
start-up	90	0.0080 (−0.15, 0.20)	5.2 (−5.2, 22)	0.88 (0.86, 0.90)
	80	0.16 (0.036, 0.39)	33 (14, 58)	1.0 (0.10, 1.0)
	70	0.32 (0.13, 0.66)	56 (29, 88)	1.2 (1.1, 1.2)
	60	0.53 (0.23, 0.99)	78 (44, 114)	1.4 (1.3, 1.5)
	50	0.79 (0.36, 1.5)	100 (61, 140)	1.7 (1.6, 1.8)
	40	1.2 (0.55, 2.1)	125 (81, 167)	2.1 (2.0, 2.3)
	30	1.7 (0.84, 3.0)	155 (108, 200)	2.8 (2.6, 3.0)
	20	2.5 (1.4, 4.6)	193 (144, 237)	4.1 (3.7, 4.4)
	10	4.5 (2.5, 8.4)	255 (202, 296)	7.0 (6.4, 7.8)
regular operation	90	0.098 (0.075, 0.13)	27 (19, 36)	0.92 (0.91, 0.93)
	80	0.18 (0.15, 0.21)	60 (50, 71)	1.0 (1.0, 1.0)
	70	0.26 (0.22, 0.30)	86 (75, 100)	1.1 (1.1, 1.1)
	60	0.35 (0.29, 0.40)	110 (98, 126)	1.3 (1.3, 1.3)
	50	0.45 (0.38, 0.52)	135 (121, 151)	1.5 (1.5, 1.5)
	40	0.59 (0.50, 0.68)	161 (146, 178)	1.8 (1.8, 1.8)
	30	0.77 (0.67, 0.90)	190 (175, 208)	2.3 (2.3, 2.4)
	20	1.1 (0.92, 1.2)	228 (212, 246)	3.3 (3.2, 3.4)
	10	1.6 (1.4, 1.9)	285 (269, 303)	5.8 (5.6, 6.0)

a The numbers in brackets represent the 95% confidence interval (2.5–97.5%).

There were no statistically significant differences in the number of consecutive flaring days between start-up and regular operations across all probabilities. However, we found a lower annual number of flaring days during start-up compared to regular operation across all probabilities, with start-up to regular operation ratios ranging from 0.19 to 0.89 (average 0.69) (Table S21). This pattern may be attributed to the smaller sample size for start-up phases relative to regular operation.

For probabilities ≤70%, the volume of gas flared per capacity was consistently higher during start-up than during regular operation, with ratios ranging from 1.2 to 2.8. In contrast, at higher probabilities (>70%) this relationship reversed, with start-up to regular operation ratios ranging from 0.082 to 0.89. Despite these trends, confidence intervals suggest true ratios may exceed 1 across all probability levels, indicating higher start-up flaring intensity cannot be ruled out even in high probability scenarios.

## 4 Discussion

### 4.1 Flaring Events Anticipated by Environmental Impact Assessments

In the commissioning and start-up years of operation, many components of LNG export facility systems are tested under different operating parameters (e.g., temperatures, pressures, flow rates), and flaring can occur fairly continuously during this phase.^54^ However, our review of environmental impact assessments (EIAs) revealed inconsistent expectations regarding the duration and intensity of start-up phase flaring from one facility to another (see Section S5.1 for references to specific EIAs).

Across all reviewed EIAs including Sabine Pass,^55^ Corpus Christi,^56^ QC LNG,^57^ Freeport LNG,^58^ Gladstone LNG,^59^ AP LNG,^27^ and PN LNG,^60^ the volumes of gas flared and associated air pollutant emissions during initial start-up were not quantified, nor were their impacts on local air quality modeled. Start-up was generally described as a short phase with negligible air pollution emissions. Our findings challenge this assumption: the start-up period can extend up to two years, and high flaring activity often persists well beyond the first year. Using probability weighted values from Table 2 and assuming a 30 year facility life, the volume of gas flared during start-up may represent between 1% (90% probability—jumping to 6% for an 80% probability) and 16% (10% probability) of total life-cycle flaring. While these percentages may appear low, flaring emits pollutants, such as benzene, that can cause acute health effects even over short exposure periods.^61^ Given the proximity of many facilities to populated residential areas, start-up emissions should be quantified in EIAs to accurately assess potential community exposure.

In contrast, some EIAs account for flaring during maintenance or short-term irregular operations. Some provide flaring volume and/or corresponding air pollutant emission estimates (Section S5.2), though these assumptions are often poorly documented.

Irregular conditions can be inferred from observations of spikes in the number of flaring days and associated increased volumes of gas flared in a given year. Our analysis shows that maintenance flaring occurs regularly, averaging 148 times per year (range 4–353, see Table S13). Importantly, we identified a strong correlation between facility capacity and annual flared volume during regular operation, suggesting that flaring could be more reliably anticipated for this phase.

Unfortunately, poor transparency in EIA assumptions makes it difficult to verify whether observed flaring events align with predicted values. While VIIRS data offer insights into flare frequency and intensity, additional information such as duration of flaring and operational context is needed to validate EIA assumptions.

Underestimating the volume of gas flared poses significant risks for nearby communities. For example, the Calcasieu Pass facility in Louisiana, U.S. (opened in 2022), has experienced chronic operational issues since its start-up phase, and is frequently flaring for multiple days or weeks. Recently, the facility applied for a new air permit to increase allowed flaring emissions,^62^ similar to the Corpus Christi facility.^63^

Despite the potential for such worst-case scenarios, including persistent start-up issues, irregular operational conditions, or even full shut-downs driven by supply demand imbalances,^64^ they are rarely disclosed or modeled in EIAs, even if they pose risks of acute pollutant exposures in nearby residential areas.^65^

### 4.2 Development of Probabilities of Occurrence of Flaring Events and Associated Volumes of Gas Flared per Life-Cycle Stage

Our study highlights significant variability in flaring patterns between facilities and across different life-cycle stages. For example, offshore facilities generally exhibit fewer flaring events, but similar total volume of gas flared. This suggests distinct operating characteristics and maintenance and possibly larger but less frequent flaring events offshore.

Start-up flaring, in particular, is difficult to anticipate, and shows no correlation with facility capacity, helping explain its frequent exclusion or underestimation in EIAs. In contrast, regular operational flaring is more consistent and closely tied to facility size, providing a firmer basis for forecasting emissions.

To address this uncertainty and support improved air pollutant emission estimates from flaring at existing export facilities worldwide, we developed probabilistic models to estimate both the likelihood of flaring events and the volumes of gas flared. These models, based on observed satellite data and fit using log-normal distributions, offer a risk-based framework for evaluating flaring across different operational stages. Rather than relying solely on the best-case scenario or average assumptions, this approach captures a range of potential outcomes, including high-impact, low probability events. This is particularly relevant in real-world examples such as Calcasieu Pass, U.S.,^62^ where unexpected operational issues have led to repeated permit applications to increase flaring thresholds. Our model provides a more robust way to anticipate and account for such variability.

It is important to note that we excluded Kiyanly LNG (Turkmenistan) from our statistical model due to its exceptionally high flaring activity compared to other facilities. While including it would have skewed our results, its exclusion also underscores a likely underestimation of flaring potential in our modeled probabilities. Kiyanly LNG should be viewed as a cautionary outlier and a reminder that extreme cases do occur and may not be well captured by current assessments. Section S4 presents the results including Kiyanly LNG.

Additionally, most LNG facilities with no detected flaring activity are relatively small. From an operational standpoint, it is highly unlikely that these facilities never flare. Instead, we see two main reasons why flaring was not detected:

1. Detection limitations—small size and timing of flares may place these facilities below the satellite detection threshold. Improved VNF algorithms and satellites may detect them in the future.
2. Environmental factors—smaller facilities are more likely to remain unobservable, particularly under persistent cloud cover.

Since our analysis is based on the ratio of flared gas volume to facility capacity, the findings may reasonably be extended to smaller facilities, but caution is warrantied, as it lacks strict empirical validation.

### 4.3 Limitations

This work is constrained by dataset limitations and methodological uncertainties. First, our analysis relied on GEM wiki^32^ capacities, since annual throughput volumes are rarely published. Ideally, flaring intensity would be reported as volume flared per actual volume of gas processed annually, rather than per maximum processing capacity.

Second, past studies (Willyard and Schade,^66^ Brandt,^67^ and Zhang et al.^68^) highlighted inconsistencies between VIIRS-based flaring estimates (i.e., VNF) and government-reported values, often provided by the operators themselves. These discrepancies can arise from both Cedigaz data used to calibrate VNF, and the self-reported figures submitted to regulators.^69^ To avoid introducing further bias, we decided not to apply post hoc corrections to reconcile these sources.

Third, while the WB and VNF datasets use the same core methodology,^42^ we observed significant discrepancies in reported flared volumes. The WB did not explain these differences. Several facilities (Wheatstone, Elba Island, Corpus Christi, APLNG, QC LNG, and Gladstone) had no reported volume of flared gas until 2020, despite consistent VNF detections, which may stem from the new VNF algorithm introduced after 2020. Spatial resolution also posed challenges: some LNG export facilities are less than 750 m apart, making flare attribution difficult. We address this by aggregating data from colocated facilities, although this may mask differences in operation behavior.

Fourth, we note that most of our start-up analysis results are based on U.S. and Australian facilities. We do not have reasons to think that the technologies used in different countries differ, but operational profiles may vary depending on regulatory requirements or company-specific practices.

Finally, it is important to recognize that VIIRS does not detect venting (unburnt gas release), which does not emit visible thermal radiation and requires specialized detection technologies. Venting has impacts on air quality and climate risks, particularly due to the high global warming potential of methane, the primary component of natural gas. Recent reports have shown large methane leaks from LNG export facilities.^63^ Estimating venting emissions requires data from high-resolution infrared sensors and satellite-based methane column measurements, which can be obtained from instruments abord aircraft, drones or satellites,^70–74^ but historical data remains limited, hindering retrospective assessments.

## Supplementary Material



## Data Availability

Code and workflow for reproducing the results are available at https://github.com/fazar37/OilGasFlaringFromVNF. Examples of input datasets are outlined within the repository. The input dataset used will be made available upon request.

## Notes

The supporting information provides information on (1) LNG export facilities included in the analysis; and (2) results (detailed information on each LNG export facility and descriptive statistics of the analyses conducted) (PDF)
