# Supplementary material for: Analysis of Flaring Activity at Liquefied Natural Gas (LNG) Export Facilities Worldwide
Source: Environ Sci Technol. 2025 Sep 20;59(38):20357–66. doi: 10.1021/acs.est.5c03755 (PMC12490014; doi:10.1021/acs.est.5c03755)
Supplement: Supplementary file 1 [file es5c03755_si_001.pdf]

Supplementary Information

Analysis of Flaring Activity at Liquefied Natural Gas (LNG) Export Facilities Worldwide

Laura Minet[1,2*], Forood Azargoshasbi[1,2], Meredith Franklin[3], Gunnar W Schade[4], Margaret J. McGregor[5], Kate McInnes[6], Tim K. Takaro[7]

[1] Department of Civil Engineering, University of Victoria, Victoria, BC, Canada, V8P 5C2
[2] Institute for Integrated Energy Systems, University of Victoria, Victoria, BC, Canada, V8P 5C2
[3] Department of Statistical Sciences and School of the Environment, University of Toronto, Toronto, ON, Canada, M5S 1A4
[4] Atmospheric Sciences, Texas A&M University, Texas, TX, US, 77843
[5] Department of Family Practice, Faculty of Medicine University of British Columbia, Vancouver, BC, Canada, V6T 1Z3
[6] Investigative Journalist & Researcher, Squamish, BC, Canada, V8B 0A1
[7] Faculty of Health Sciences, Simon Fraser University, Burnaby, BC, Canada, V5A 1S6

[*] Corresponding author: 3800 Finnerty Road, Victoria, BC V8P 5C2, Canada; 250-7231-8987; lauraminet@uvic.ca

Number of pages: 50

Number of figures: 18

Number of tables: 32

S1. Methodology

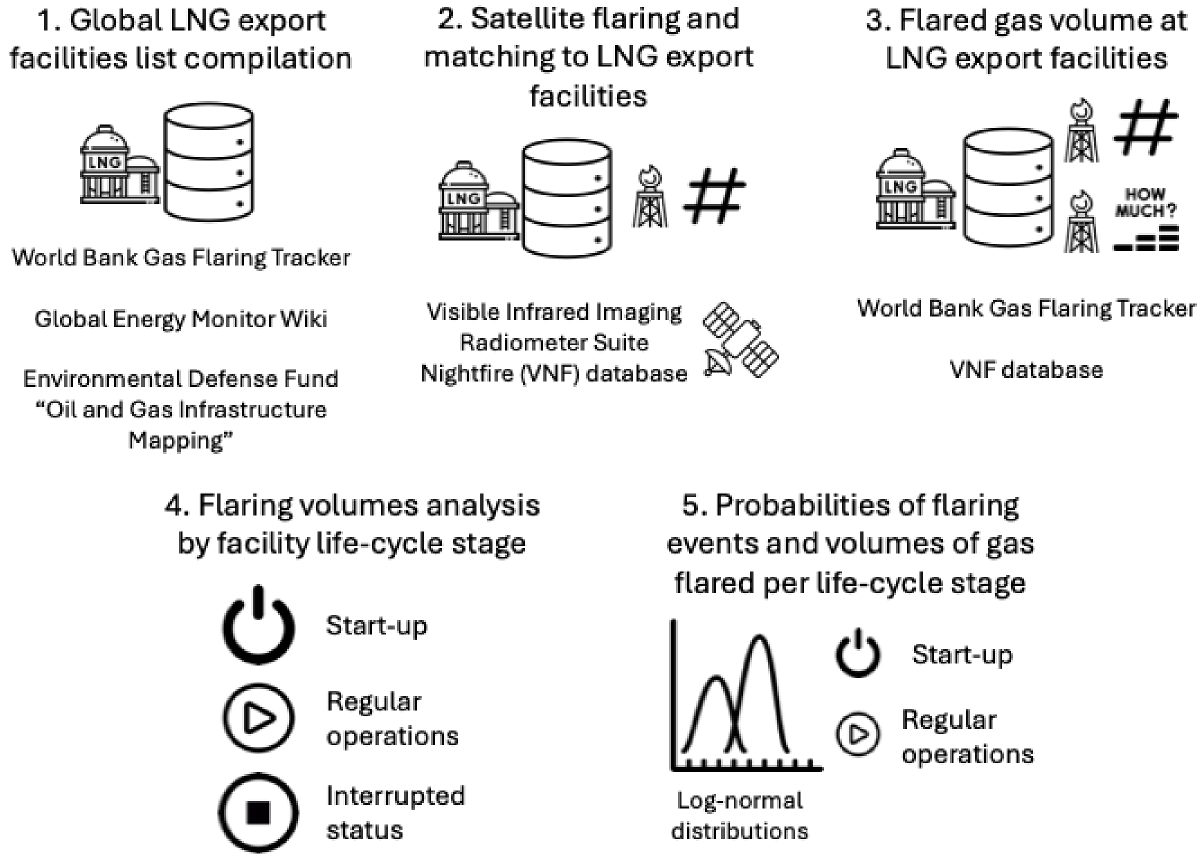

Figure S1. Diagram of the five steps of our methodology

S2. Analysis of the 61 LNG Export Facilities Identified

S2.1. Characteristics of the Facilities

S2.1.1. Location

Figure S2 identifies the location of the 61 LNG export facilities included in the dataset. Descriptive statistics on the flaring data recorded for each facility during the 2012-2022 period are provided in Table S1.

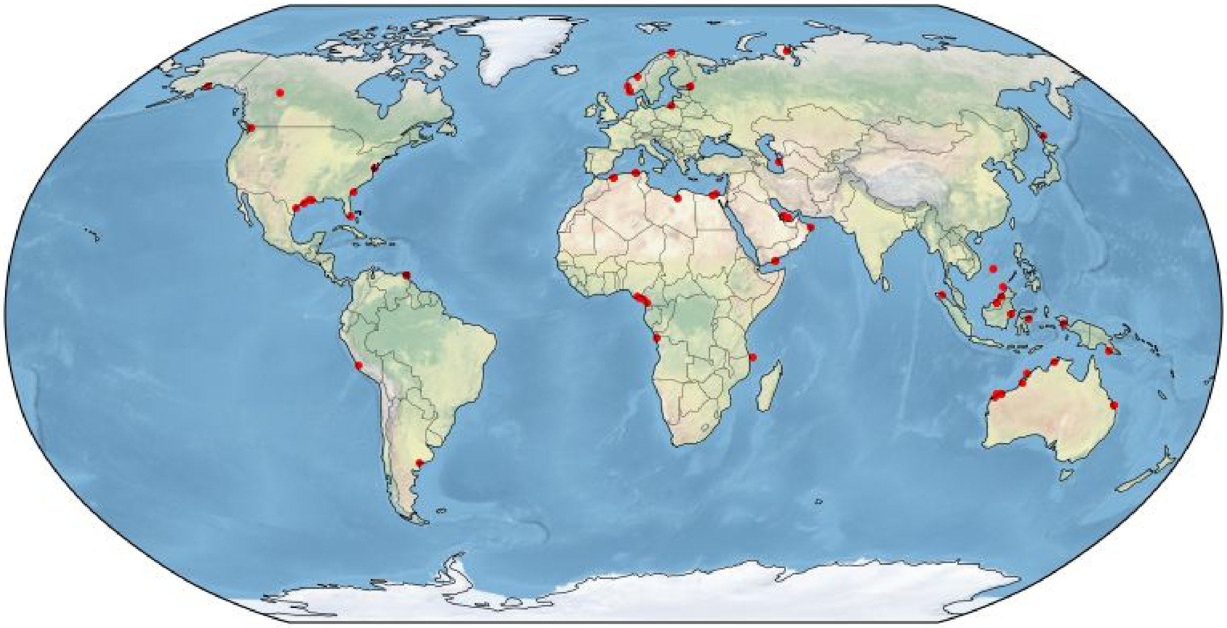

Figure S2. Location of the 61 LNG export facilities identified

S2.1.2. Capacities

Table S2 details the yearly capacities of the 61 LNG export facilities identified globally.

Table S1. Summary of the 61 LNG export facilities, with average flaring data provided for 2012-2022. mtpa and bcm stand for million tonnes per annum and billion cubic metres, respectively. N/A means no flaring data was available in the VNF dataset.

	Facility Name	Country	Onshore / Offshore	Start Year	Capacity range since 2012 (mtpa)	Average number of flaring days per year since opening	Average volume of gas flared (bcm)	Average volume of gas flared per capacity of the facility (% - bcm/bcm)
**Facilities commissioned before 2012**	Tilbury Island LNG	Canada	On	1971	0.03 - 0.28	N/A	N/A	N/A
	Brunei LNG	Brunei Darussalam	On	1973	7.2 - 7.2	119	0.058	0.8
	AB LNG	Algeria	On	1981	16.1- 20.8	320	0.54	2.8
	MLNG	Malaysia	On	1995	18 - 18	137	0.11	0.25
	DM LNG		On	1995	9.6 - 9.6			
	SM LNG		On	1983	8.4 - 8.4			
	TM LNG		On	2003	7.2 - 7.2			
	ADNOC LNG	UAE	On	1994	7.6 - 7.6	319	0.15	2.03
	Bontang LNG	Indonesia	On	1998	11.5 - 21	197	0.11	0.58
	Kollsnes LNG	Norway	On	2003	0.12 - 0.12	3	N/A	N/A
	Snurrevarden LNG	Norway	On	2003	0.02 - 0.02	N/A	N/A	N/A
	Damietta LNG	Egypt	On	2005	1.0 - 5.0	111	0.022	1.52
	ELNG	Egypt	On	2005	7.2 - 7.2	229	0.066	0.91
	Darwin LNG	Australia	On	2006	3.7 - 3.7	33	0.017	0.47
	Qalhat LNG	Oman	On	2006	10.4 - 10.4	126	0.026	0.25
	PE LNG	Equatorial Guinea	On	2007	3.7 - 3.7	72	0.025	0.68
	Snohvit	Norway	Off	2007	4.2 - 4.2	N/A	N/A	N/A
	Atlantic LNG	Trinidad and tobago	On	2007	12.0 - 12.0	220	0.092	0.76
	North West Shelf	Australia	On	2008	16.3 - 16.3	161	0.066	0.41
	NLNG	Nigeria	On	2008	22.0 - 22.0	162	0.13	0.59
	Tangguh LNG	Indonesia	On	2009	7.6 - 7.6	74	0.044	0.58

	Sakhalin-2 LNG	Russia	On	2009	10.0 - 11.5	90	0.022	0.21
	Kiyanly LNG	Turkmenistan	On	2009	0.2 - 0.2	259	0.11	56
	Risavika LNG	Norway	On	2010	0.33 - 0.33	2	N/A	N/A
	Peru LNG	Peru	On	2010	4.45 - 4.45	21	0.0037	0.08
	Rasgas	Qatar	On	2010	36.3 - 36.3	280	0.11	0.32
	Qatargas	Qatar	On	2011	41.1 - 41.1	330	0.14	0.34
**Facilities commissioned after 2012**	Angola LNG	Angola	On	2012	5.2 - 5.2	119	0.099	1.9
	Pluto LNG	Australia	Off	2012	4.9 - 4.9	95	0.052	1.05
	Skikda	Algeria	On	2013	4.5 - 4.5	277	0.12	2.57
	PNG LNG	Papua New Guinea	On	2013	8.3 - 8.3	58	0.035	0.42
	AP LNG	Australia	On	2016	9 - 9	157	0.039	0.14
	Gladstone		On	2015	5 - 10			
	QC LNG		On	2015	8.5 - 8.5			
	DS LNG	Indonesia	On	2015	2.0 - 2.0	23	0.013	0.65
	Sabine Pass LNG	U.S.	On	2015	10.0 - 30.0	112	0.040	0.22
	Gorgon LNG	Australia	On	2016	10.4 - 15.6	93	0.029	0.23
	Hialeah LNG	U.S.	On	2016	0.06 - 0.06	N/A	N/A	N/A
	Wheatstone LNG	Australia	On	2017	4.45 - 8.9	151	0.054	0.6
	Satu FLNG	Malaysia	Off	2017	1.2 - 1.2	N/A	N/A	N/A
	Yamal LNG	Russia	On	2017	5.5 - 17.4	191	0.12	0.99
	Ichthys FLNG	Australia	Off	2018	8.9 - 8.9	31	N/A	N/A
	Cameroon FLNG	Cameroon	Off	2018	2.4 - 2.4	25	0.0098	0.41
	Corpus LNG	U.S.	On	2018	5.0 - 15.0	95	0.020	0.15
	Cove Point LNG	U.S.	On	2018	5.3 - 5.3	14	0.0018	0.03
	Prelude FLNG	Australia	Off	2019	3.6 - 3.6	228	0.13	3.7
	Greenville LNG	Nigeria	On	2019	0.82 - 0.82	20	0.0028	0.34
	Vysotsk LNG	Russia	On	2019	0.66 – 0.66	35	0.0052	0.42
	PO LNG		On	2022	1.5-1.5			
	Cameron LNG	U.S.	On	2019	4.5 - 13.5	85	0.022	0.25
	Elba Island LNG	U.S.	On	2019	12.5 - 25.0	6	0.001	0

	Freeport LNG	U.S.	On	2019	15.0 - 15.0	N/A	N/A	N/A
	Fort Nelson LNG	Canada	On	2021	0.02 - 0.02	N/A	N/A	N/A
	DF LNG	Malaysia	Off	2021	1.5 - 1.5	N/A	N/A	N/A
	CS FLNG	Mozambique	Off	2022	3.4 - 3.4	167	0.43	13
	Calcasieu Pass	U.S.	On	2022	12.0 - 12.0	86	0.017	0.14
**Facilities with irregular operations**	Kenai LNG	U.S.	On	1969	1.5 - 1.5	7	0.0024	0.16
	Marsa LNG	Libya	On	1970	3.2 - 3.2	321	0.24	7.5
	Tjelbergodden LNG	Norway	On	1997	0.01 - 0.01	2	N/A	N/A
	Yemen LNG	Yemen	On	2010	6.7 - 7.2	223	0.05	0.73
	Bahia Blanca FLNG	Argentina	On	2019	0.45 - 0.45	112	0.021	4.6

Table S2. Yearly capacities of the 61 LNG export facilities (in million tonnes per annum - mtpa)

Facility	2012	2013	2014	2015	2016	2017	2018	2019	2020	2021	2022
**AB LNG**	16.14	16.14	20.84	20.84	20.84	20.84	20.84	20.84	20.84	20.84	20.84
**ADNOC LNG**	7.6	7.6	7.6	7.6	7.6	7.6	7.6	7.6	7.6	7.6	7.6
**Angola LNG**	5.2	5.2	5.2	5.2	5.2	5.2	5.2	5.2	5.2	5.2	5.2
**AP LNG, Gladstone, QC LNG**				13.5	27.5	27.5	27.5	27.5	27.5	27.5	27.5
**Atlantic LNG**	12	12	12	12	12	12	12	12	12	12	12
**Bahia Blanca FLNG**								0.45	0.45	0.45	0.45
**Bontang LNG**	21	21	21	21	21	21	21	21	11.5	11.5	11.5
**Brunei LNG**	7.2	7.2	7.2	7.2	7.2	7.2	7.2	7.2	7.2	7.2	7.2
**Calcasieu Pass**											12
**Cameron LNG**								4.5	13.5	13.5	13.5
**Cameroon FLNG**							2.4	2.4	2.4	2.4	2.4
**Corpus LNG**							5	10	15	15	15
**Cove Point LNG**							5.3	5.3	5.3	5.3	5.3
**CS FLNG**											3.4
**Damietta LNG**	1	1	1	1	1	1	1	1	1	5	5
**Darwin LNG**	3.7	3.7	3.7	3.7	3.7	3.7	3.7	3.7	3.7	3.7	3.7
**DF LNG**										1.5	1.5
**DS LNG**				2	2	2	2	2	2	2	2
**Elba Island LNG**								12.5	25	25	25
**ELNG**	7.2	7.2	7.2	7.2	7.2	7.2	7.2	7.2	7.2	7.2	7.2
**Fort Nelson LNG**										0.02	0.02
**Freeport LNG**								15	15	15	15
**Gorgon LNG**					10.4	15.6	15.6	15.6	15.6	15.6	15.6
**Greenville LNG**								0.82	0.82	0.82	0.82
**Hialeah LNG**					0.06	0.06	0.06	0.06	0.06	0.06	0.06
**Ichthys FLNG**							8.9	8.9	8.9	8.9	8.9

**Kenai LNG**	1.5	1.5	1.5	1.5	1.5	1.5	1.5	1.5	1.5	1.5	1.5
**Kiyanly LNG**	0.2	0.2	0.2	0.2	0.2	0.2	0.2	0.2	0.2	0.2	0.2
**Kollsnes LNG**	0.12	0.12	0.12	0.12	0.12	0.12	0.12	0.12	0.12	0.12	0.12
**Marsa LNG**	3.2	3.2	3.2	3.2	3.2	3.2	3.2	3.2	3.2	3.2	3.2
**MLNG, DM LNG, SM LNG, TM LNG**	43.2	43.2	43.2	43.2	43.2	43.2	43.2	43.2	43.2	43.2	43.2
**NLNG**	22	22	22	22	22	22	22	22	22	22	22
**North West Shelf**	16.3	16.3	16.3	16.3	16.3	16.3	16.3	16.3	16.3	16.3	16.3
**PE LNG**	3.7	3.7	3.7	3.7	3.7	3.7	3.7	3.7	3.7	3.7	3.7
**Peru LNG**	4.45	4.45	4.45	4.45	4.45	4.45	4.45	4.45	4.45	4.45	4.45
**Pluto LNG**	4.9	4.9	4.9	4.9	4.9	4.9	4.9	4.9	4.9	4.9	4.9
**PNG LNG**		8.3	8.3	8.3	8.3	8.3	8.3	8.3	8.3	8.3	8.3
**Prelude FLNG**								3.6	3.6	3.6	3.6
**Qalhat LNG**	10.4	10.4	10.4	10.4	10.4	10.4	10.4	10.4	10.4	10.4	10.4
**Qatargas**	41.1	41.1	41.1	41.1	41.1	41.1	41.1	41.1	41.1	41.1	41.1
**Rasgas**	36.3	36.3	36.3	36.3	36.3	36.3	36.3	36.3	36.3	36.3	36.3
**Risavika LNG**	0.33	0.33	0.33	0.33	0.33	0.33	0.33	0.33	0.33	0.33	0.33
**Sabine Pass LNG**				10	10	20	25	25	25	30	30
**Sakhalin-2 LNG**	10	10	10	10	10	10	10	10	10	10.8	11.5
**Satu FLNG**						1.2	1.2	1.2	1.2	1.2	1.2
**Skikda**		4.5	4.5	4.5	4.5	4.5	4.5	4.5	4.5	4.5	4.5
**Snohvit**	4.2	4.2	4.2	4.2	4.2	4.2	4.2	4.2	4.2	4.2	4.2
**Snurrevarden LNG**	0.02	0.02	0.02	0.02	0.02	0.02	0.02	0.02	0.02	0.02	0.02
**Tangguh LNG**	7.6	7.6	7.6	7.6	7.6	7.6	7.6	7.6	7.6	7.6	7.6
**Tilbury Island LNG**	0.03	0.03	0.03	0.03	0.03	0.03	0.28	0.28	0.28	0.28	0.28
**Tjelbergodden LNG**	0.01	0.01	0.01	0.01	0.01	0.01	0.01	0.01	0.01	0.01	0.01
**Vysotsk LNG, PO LNG**								0.66	0.66	0.66	2.16
**Wheatstone LNG**						4.45	8.9	8.9	8.9	8.9	8.9
**Yamal LNG**						5.5	16.5	16.5	16.5	17.4	17.4
**Yemen LNG**	6.7	6.7	6.7	6.7	6.7	7.2	7.2	7.2	7.2	7.2	7.2

S2.2. Cloud Mask Sensitivity Analysis

VNF recommends removing data associated with cloudy conditions and later adjusting for missing days because the VIIRS might not detect any flaring activity on cloudy days. There should not be any risk of false detection of flares (i.e., detecting a flare on a cloudy day that is actually not a flare). This means that our approach of including all data regardless of the cloud masks is at worst underestimating the number of days with flaring. Nevertheless, we conduct here a sensitivity analysis and compare the annual number of flares considered for each facility with three methods.

The total number of days with flares (i.e., temperature higher than 1,100 K) recorded by the VIIRS within 750 m of a facility is equal to the sum of $N_{flaring,\ clear}$ (number of days with a flare with clear sky) and $N_{flaring,\ cloudy}$ (number of days with a flare with cloudy sky). The three methods proposed for determining the annual number of days with flares are:

1) $N_{flaring,\ clear} + N_{flaring,\ cloudy}$: this means we include all days, regardless of the cloud mask (results presented in the main manuscript);
2) $N_{flaring,\ clear}$: this means we include only days with clear sky. This most certainly underestimates the annual number of flares considered since flaring can occur on cloudy days;
3) Equation (1): this method adapts the proportionality approach to ensure that the total number of days with flares is lower than the sum of $N_{flaring,\ clear}$ and $N_{flaring,\ cloudy}$.

$$\#_{Adjusted} = \begin{cases} \#_{ClearSky} + \frac{(\#_{Total} - \#_{ClearSky}) * \#_{ClearSky}}{\#_{DaysMonth}} \ if \ \#_{Adjusted} \leq \#_{DaysMonth} \\ \#_{Total} \ if \ \#_{ClearSky} + \frac{(\#_{Total} - \#_{ClearSky}) * \#_{ClearSky}}{\#_{DaysMonth}} \geq \#_{Total} \\ \#_{DaysMonth} \ if \ \#_{ClearSky} + \frac{(\#_{Total} - \#_{ClearSky}) * \#_{ClearSky}}{\#_{DaysMonth}} \geq \#_{DaysMonth} \end{cases} \quad (1)$$

where:

$\#_{Adjusted}$ is the adjusted number of flaring days in a given month

$\#_{ClearSky}$ is the number of flaring days detected by VIIRS under clear sky conditions in a month

$\#_{Total}$ is the total number of flaring days detected by VIIRS in a month

$\#_{DaysMonth}$ is the total number of days in the month

Tables S3 and S4 provides percentage differences in number of flaring days calculated following Equations (S2) and (S3), where AnnualCount represents the annual number of flaring days detected with each method: allData includes both clear and cloudy sky days (method 1, reported in the main manuscript); ClearSky includes only clear days (method 2); and ClearSkyAdjusted corresponds to method 3.

$$Percentage\ Difference = \frac{AnnualCount_{allData} - AnnualCount_{ClearSky}}{AnnualCount_{allData}} \times 100 \qquad (S2)$$

$$Percentage\ Difference = \frac{AnnualCount_{allData} - AnnualCount_{ClearSkyAdjusted}}{AnnualCount_{allData}} \times 100 \qquad (S3)$$

For some facilities, we observe large differences, meaning that temperatures higher than 1,100 K were detected by the VIIRS on many days, but were filtered out by the cloudy sky condition. Those high temperatures are unlikely to be associated with sun radiation reflection by clouds since the observation by VIIRS happened during the night.

Table S5 provides the total number of flaring days per year for all facilities and all years, as considered in the main manuscript.

Table S3. Percentage difference in the yearly number of flaring days detected under a clear sky condition, and the yearly number of flaring days detected during both cloudy and clear sky days

Facilities	2012	2013	2014	2015	2016	2017	2018	2019	2020	2021	2022	Average	Total over 2012-2022
**AB LNG**	21.5	19.8	18.1	16.9	15.5	20.5	31	28.4	32.9	34.1	39.9	25.3	892
**ADNOC LNG**	15.6	9.1	8.8	13.1	10.9	34.3	59.9	63.7	36.7	29.9	33.2	28.7	1021
**Angola LNG**	22.4	16.1	29.9	21.4	16.5	29.3	48.5	48.2	45.5	62	72	37.4	459
**AP LNG, Gladstone, QC LNG**				10.6	8.6	30.9	25.4	27.1	25.6	31.1	38.4	24.7	278
**Atlantic LNG**	27.8	15.9	15.6	17.5	18.8	32.8	50.7	57.3	50.2	53.3	50	35.5	887
**Bahia Blanca FLNG**								47.1	22.4	18.9	25	28.3	129
**Bontang LNG**	29.3	28.4	31	20.4	24.8	51.4	67.1	52.2	77	94.5	91.2	51.6	1079
**Brunei LNG**	55.7	58.9	43.7	41.4	41	65.3	70.5	63.8	76.2	98.3	98.1	64.8	829
**Calcasieu Pass**											65.1	65.1	56
**Cameron LNG**								39.6	41.5	26.7	24.2	33	124
**Cameroon FLNG**							68.1	85	70	91.7		78.7	82
**Corpus LNG**							37.2	42.1	21.4	32	28.6	32.3	145
**Cove Point LNG**							37.5	33.3	53.8	10	25	31.9	25
**CS FLNG**											47.9	47.9	80
**Damietta LNG**	2.7	7	6.8	4.9	4.8	23.5	7.5	8	5.6	13.6	10.7	8.6	111
**Darwin LNG**	23.8	50	0	50	0	75	33.7	46.3	18	43.6	47.1	35.2	124
**DS LNG**				5	42.3	70	84.6	83.3	81.2			61.1	71
**Elba Island LNG**								33.3	27.3	40		33.5	6
**ELNG**	5	4.7	4.7	8.8	6.4	13.1	6.5	12.4	11.5	18.1	12.6	9.4	254
**Gorgon LNG**					5.9	34.7	26.1	34.1	22.1	19.7	18.6	23	148
**Greenville LNG**								42.1	50	40.6	38.1	42.7	33
**Kenai LNG**		0		20	11.8	33.3						16.3	5
**Kiyanly LNG**	23.4	19.7	22	22.4	17.6	21.7	22.9	18.5	35.5	48	44.4	26.9	773
**Marsa LNG**	8.1	9	9	9.5	9.9	20.4	36.5	29.5	14.7	13.7	17	16.1	567
**MLNG, DM LNG, SM LNG, TM LNG**	59.4	60.2	49.2	50.8	51.9	75.4	81.9	81.8	83.2	99.2		69.3	950
**NLNG**	36	40.6	35.1	30.1	29.4	45.5	62.2	63.9	71.9	92.4	88.3	54.1	998

**North West Shelf**	7.6	12.1	10	13.6	5.2	36	40.4	45.4	12.9	17.4	22	20.2	389
**PE LNG**	52.2	48.1	44.4	38.3	39.7	56.7	47.4	65.8	70.1	91	83.3	57.9	473
**Peru LNG**	33.3	0	12.5	5.6	23.1	0	21.1	35.3	27.8	35.1	48.6	22	67
**Pluto LNG**	9.2	10.2	8.1	14	10	35.2	28.3	34.1	27.8	30.5	26.8	21.3	184
**PNG LNG**		25	40.1	28	26.3	47.1	60	40.6	50.9	67.9	77.3	46.3	314
**Prelude FLNG**								30.7	30.4	36.6	52.6	37.6	328
**Qalhat LNG**	15.9	5.5	5.1	8.9	4.3	22.8	28.8	31.7	27.2	14	11.6	16	241
**Qatargas**	14.4	11.4	9.6	8.7	9.5	23.9	35.9	56.3	29.5	17.5	23	21.8	792
**Rasgas**	18.9	11.5	9.6	9.8	10.2	20.8	34.2	43.9	27	17.1	23.1	20.6	598
**Sabine Pass LNG**				20	14.6	35.9	47.4	32.8	31.7	32.2	31.5	30.8	290
**Sakhalin-2 LNG**	26.1	27	16.9	19	27.3	38.6	38.1	40.9	42	61.4	71.6	37.2	420
**Skikda**		21	15.8	16.4	15.2	24.7	44.8	47.7	26.3	35.4	35.8	28.3	790
**Tangguh LNG**	54.5	42.9	44.4	37.8	36.4	55.8	61.3	61.7	76.2	97.2	99.2	60.7	565
**Vysotsk LNG, PO LNG**								28.3	23.5	71.4	75.7	49.7	75
**Wheatstone LNG**						35.2	41	33.5	17.2	11.5	17.4	26	270
**Yamal LNG**						20.4	30.2	20.9	48.3	66.7	67.9	42.4	505
**Yemen LNG**	18.8	9.8	10.5	11.4	11.6	9.9	22.1	19	19	12.6	4.8	13.6	353

Table S4. Percentage difference in the yearly number of flaring days detected under a clear sky condition and adjusted with method 3 (equation 1), and the yearly number of flaring days detected during both cloudy and clear sky days

**Facilities**	**2012**	**2013**	**2014**	**2015**	**2016**	**2017**	**2018**	**2019**	**2020**	**2021**	**2022**	**Average**	**Total over 2012-2022**
**AB LNG**	8.7	5.4	4.9	4.2	4	6.9	12.1	9.9	12.2	14.6	19.5	9.3	327
**ADNOC LNG**	5.3	1.5	1.9	3.7	2.2	16.2	37.2	43.2	15.5	10.3	13.7	13.7	490
**Angola LNG**	18.8	9.1	22.8	16.1	9.3	25.9	41.7	40.4	37.2	54.6	66	31.1	372
**AP LNG, Gladstone, QC LNG**				4.1	4.3	25.5	16	19.4	18.8	23.8	30.4	17.8	192
**Atlantic LNG**	16.7	7.1	7.6	8.3	12.3	22	32	38.1	32.5	38.8	38.1	23.1	573
**Bahia Blanca FLNG**								38.7	17.2	14.2	19.4	22.4	102
**Bontang LNG**	18.6	18.8	18.6	9.8	14.4	39.9	53	35.5	67.5	92.1	87.8	41.4	849

**Brunei LNG**	49.5	53.4	34.9	29.9	32.1	57.9	63.6	55.9	71.4	97.5	97.1	58.5	741
**Calcasieu Pass**											59.3	59.3	51
**Cameron LNG**								32.4	32.6	23.3	21.2	27.4	101
**Cameroon FLNG**							63.8	85	70	91.7		77.6	79
**Corpus LNG**							34.9	35.8	13.8	24.2	26.5	27.1	115
**Cove Point LNG**							34.4	33.3	53.8	10	25	31.3	24
**CS FLNG**											36.5	36.5	61
**Damietta LNG**	1.8	5.6	4.1	4.9	4.8	23.5	5.7	4.9	5.6	4.8	3.7	6.3	56
**Darwin LNG**	23.8	50	0	50	0	75	28.1	40.2	14.8	41	44.1	33.4	110
**DS LNG**				5	42.3	70	84.6	83.3	81.2			61.1	71
**Elba Island LNG**								33.3	27.3	40		33.5	6
**ELNG**	2.5	2.3	1.9	4.9	3.8	9.2	2.4	3.3	2.9	5.5	3.7	3.9	93
**Gorgon LNG**					3.9	30.6	18.3	28.6	18.6	16.4	14.4	18.7	118
**Greenville LNG**								42.1	50	37.5	38.1	41.9	32
**Kenai LNG**		0		20	11.8	33.3						16.3	5
**Kiyanly LNG**	11.9	8.3	8.4	10	7.8	8.4	11.2	9.3	19.1	29.1	26.3	13.6	391
**Marsa LNG**	2.6	1.9	1.5	1.8	1.8	6.2	16.8	11.3	2.9	3	5.1	5	174
**MLNG, DM LNG, SM LNG, TM LNG**	53.1	54.1	40.7	38.7	41	68.9	75.8	74.6	77.6	99.2		62.4	851
**NLNG**	30	31.2	25.3	21.6	20.2	34.6	50	51.5	61.6	88.6	84	45.3	839
**North West Shelf**	4.4	4.7	5.3	8.4	3.4	22.1	25.6	32.8	9.7	14	17.6	13.4	249
**PE LNG**	50	44.4	41.7	36.2	36.2	52.2	38.5	59	65.5	89.7	80.3	54	436
**Peru LNG**	33.3	0	12.5	5.6	23.1	0	21.1	35.3	27.8	29.9	45.7	21.3	62
**Pluto LNG**	3	5.7	6.8	10.8	8	31	25	28.6	24.1	27.1	24.4	17.7	139
**PNG LNG**		25	31.4	28	26.3	47.1	54.1	37.5	47.2	61.3	72.2	43	282
**Prelude FLNG**								13.5	13.3	28.2	39.5	23.6	191
**Qalhat LNG**	13	4.4	4	6.7	2.6	15.4	20.9	23.4	17.8	8.1	7.5	11.3	167
**Qatargas**	4.9	2	1.8	1.5	1.5	8.2	14.4	34.3	9.3	3.8	7.6	8.1	294
**Rasgas**	7	2.1	1.8	1.8	2.2	8.6	17.5	27.5	13.7	7.2	14	9.4	261
**Sabine Pass LNG**				20	11.5	30.8	38.3	25.2	22.4	23.8	23.8	24.5	224
**Sakhalin-2 LNG**	26.1	22.5	15.3	15.9	23.6	34.3	32	30.5	34.8	53.5	63.8	32	357

**Skikda**		7.9	5.1	6.5	6.1	12.6	25.2	28.3	10.3	17.5	19.2	13.9	386
**Tangguh LNG**	50.9	39.3	42.2	35.1	33.3	51.9	54.8	54.2	70.5	96.3	99.2	57.1	534
**Vysotsk LNG, PO LNG**								26.1	23.5	71.4	72.9	48.5	72
**Wheatstone LNG**						24.8	23.1	23.6	14	8.2	14.1	18	178
**Yamal LNG**						15.7	17.3	11.9	34.5	53.2	56.7	31.6	374
**Yemen LNG**	9.6	4.7	2.6	3	4.8	7.2	8.7	8.3	9.1	6.5	3.6	6.2	151

Table S5. Yearly number of flaring days for each facility

**Facilities**	**2012**	**2013**	**2014**	**2015**	**2016**	**2017**	**2018**	**2019**	**2020**	**2021**	**2022**	**Total over 2012-2022**
**AB LNG**	275	334	326	332	323	303	323	335	343	314	313	3521
**ADNOC LNG**	282	330	320	297	322	297	344	322	335	341	322	3512
**Angola LNG**	85	186	127	112	194	58	103	114	121	108	100	1308
**AP LNG, Gladstone, QC LNG**				246	209	94	181	144	133	122	125	1254
**Atlantic LNG**	198	239	224	228	154	177	272	286	255	214	176	2423
**Bahia Blanca FLNG**								119	116	106	108	449
**Bontang LNG**	188	176	210	235	202	173	234	245	191	164	148	2166
**Brunei LNG**	97	73	126	174	134	121	129	127	105	120	103	1309
**Calcasieu Pass**											86	86
**Cameron LNG**								111	135	60	33	339
**Cameroon FLNG**							69	20	10	12	14	125
**Corpus LNG**							43	95	159	128	49	474
**Cove Point LNG**							32	9	13	10	8	72
**CS FLNG**											167	167
**Damietta LNG**	112	71	148	41	21	17	53	163	54	273	271	1224
**Darwin LNG**	21	4	20	4	4	4	89	82	61	39	34	362
**DS LNG**				60	26	10	26	18	16	24	7	187
**Elba Island LNG**								3	11	5	0	19
**ELNG**	199	171	214	182	156	130	246	306	313	310	294	2521

**Gorgon LNG**					102	72	142	91	86	61	97	651
**Greenville LNG**								19	8	32	21	80
**Kenai LNG**	0	1	0	5	17	6	0	0	0	0	0	29
**Marsa LNG**	273	323	334	328	333	323	310	319	341	335	311	3530
**MLNG, DM LNG, SM LNG, TM LNG**	96	98	118	181	156	122	149	181	143	123	141	1508
**NLNG**	100	138	154	153	163	156	188	194	185	184	162	1777
**North West Shelf**	158	257	190	154	116	222	223	183	93	86	91	1773
**PE LNG**	46	54	36	47	58	67	135	117	87	78	66	791
**Peru LNG**	9	8	8	18	13	6	19	17	18	77	35	228
**Pluto LNG**	271	176	74	93	50	71	60	91	54	59	41	1040
**PNG LNG**		4	137	25	19	17	85	32	53	106	97	575
**Prelude FLNG**								296	293	131	190	910
**Qalhat LNG**	69	91	99	90	115	149	139	145	169	172	146	1384
**Qatargas**	285	343	334	334	337	318	340	327	353	343	317	3631
**Rasgas**	286	339	334	327	324	269	269	244	248	251	186	3077
**Sabine Pass LNG**				20	96	78	133	131	161	143	130	892
**Sakhalin-2 LNG**	23	89	59	63	55	70	97	154	112	127	141	990
**Skikda**		290	297	262	263	239	290	283	300	285	265	2774
**Tangguh LNG**	55	28	45	37	33	52	93	120	122	107	121	813
**Vysotsk LNG, PO LNG**								46	17	7	70	140
**Wheatstone LNG**						165	273	161	93	122	92	906
**Yamal LNG**						108	225	201	203	222	187	1146
**Yemen LNG**	218	215	304	299	249	111	289	253	231	199	84	2452

S2.3. Volumes of Gas Flared at each Facility

S2.3.1. Comparison Between VNF and WB Datasets for Volume of Gas Flared

Figure S3 compares the data extracted from the VNF and WB datasets, with facilities grouped by country. Table S6 compares the volumes of gas flared provided in the VNF and WB datasets. Table S7 provides percentage differences, calculated as in equation (S4), where AnnualVolume represents the annual volume of gas flared reported by each dataset (VNF and WB).

$$Percentage\ Difference = \frac{AnnualVolume_{VNF} - AnnualVolume_{WB}}{AnnualVolume_{VNF}} \times 100 \qquad (S4)$$

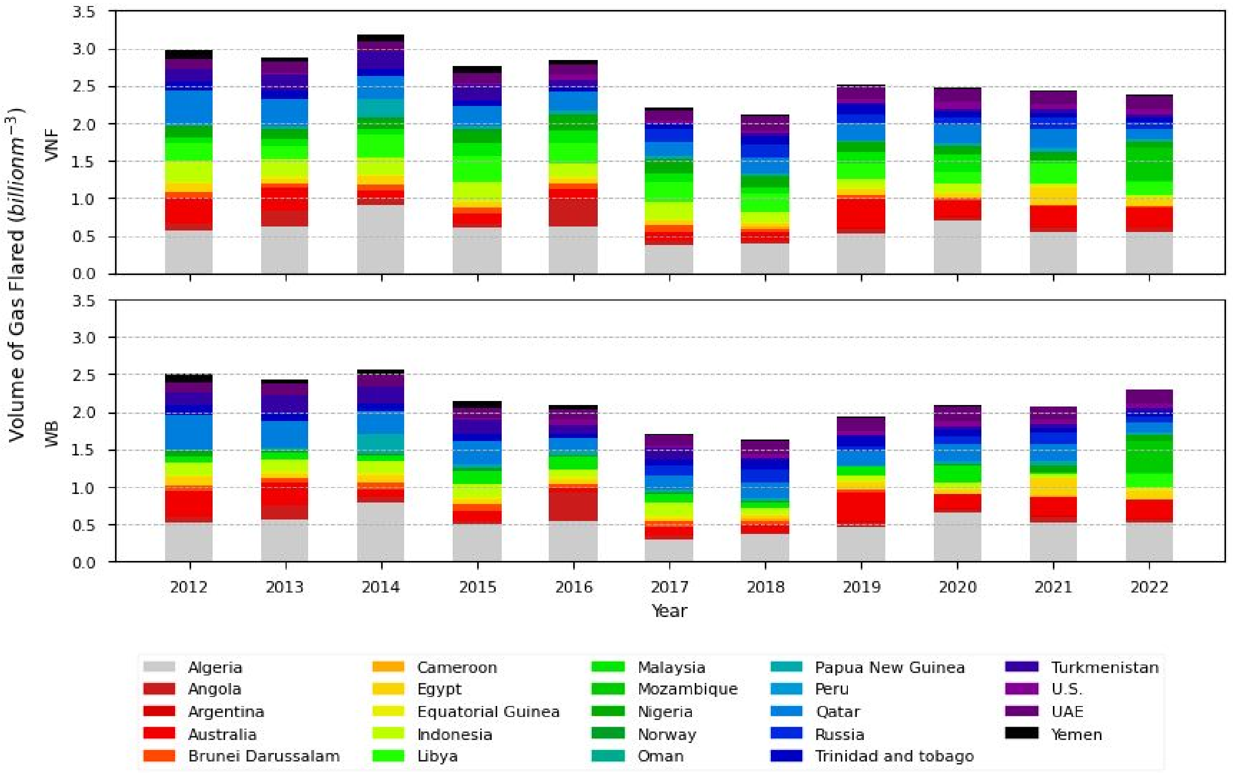

Figure S3. Comparison of the annual volumes of gas flared by LNG export facilities grouped by country estimated by the World Bank (WB) and VIIRS Night Fire (VNF) datasets

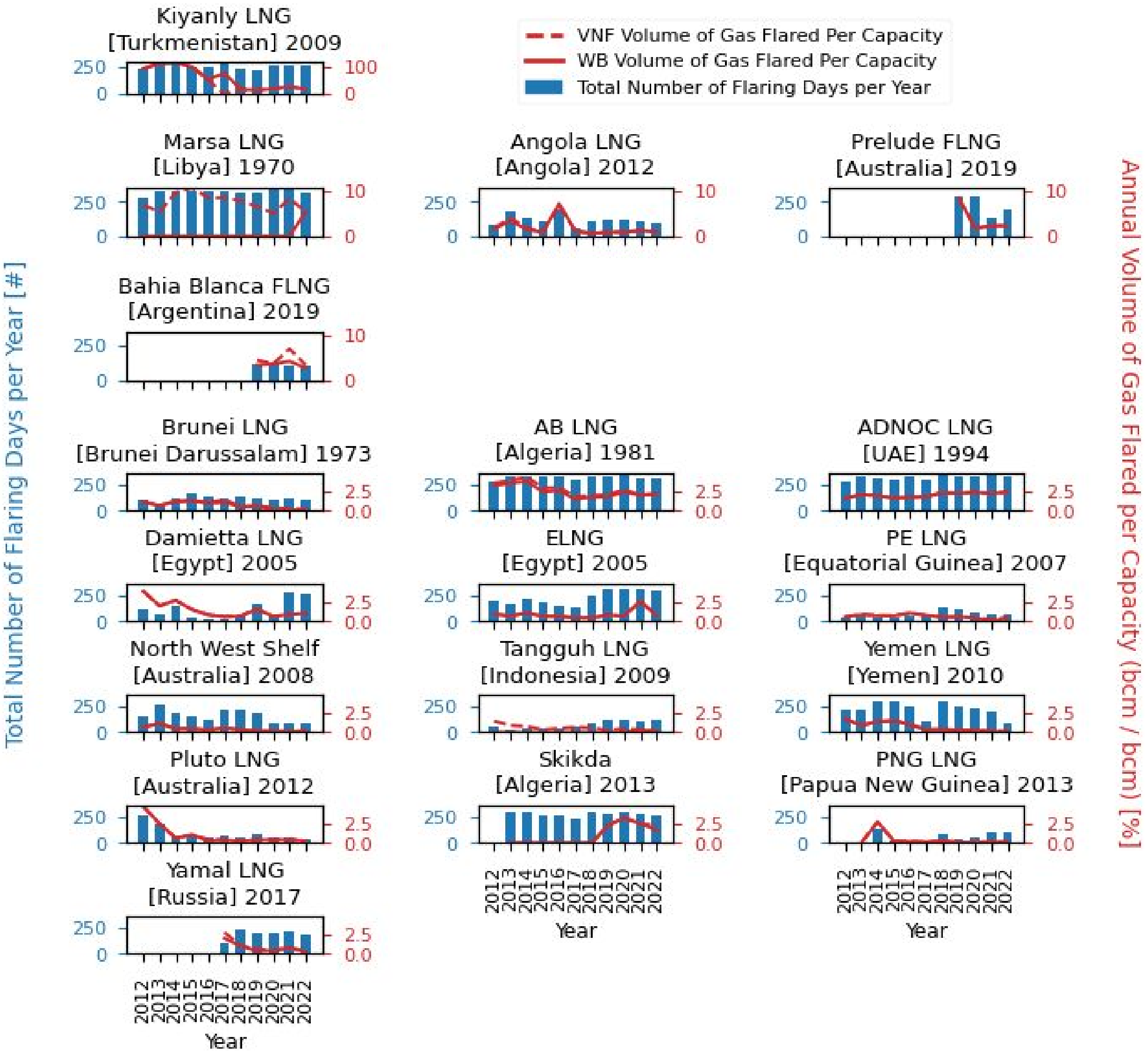

VNF Volume of Gas Flared Per Capacity
WB Volume of Gas Flared Per Capacity
Total Number of Flaring Days per Year
Kiyanly LNG [Turkmenistan] 2009
Marsa LNG [Libya] 1970
Angola LNG [Angola] 2012
Prelude FLNG [Australia] 2019
Bahia Blanca FLNG [Argentina] 2019
Brunei LNG [Brunei Darussalam] 1973
AB LNG [Algeria] 1981
ADNOC LNG [UAE] 1994
Damietta LNG [Egypt] 2005
ELNG [Egypt] 2005
PE LNG [Equatorial Guinea] 2007
North West Shelf [Australia] 2008
Tangguh LNG [Indonesia] 2009
Yemen LNG [Yemen] 2010
Pluto LNG [Australia] 2012
Skikda [Algeria] 2013
PNG LNG [Papua New Guinea] 2013
Yamal LNG [Russia] 2017
Total Number of Flaring Days per Year [#]
Annual Volume of Gas Flared per Capacity (bcm / bcm) [%]
Year

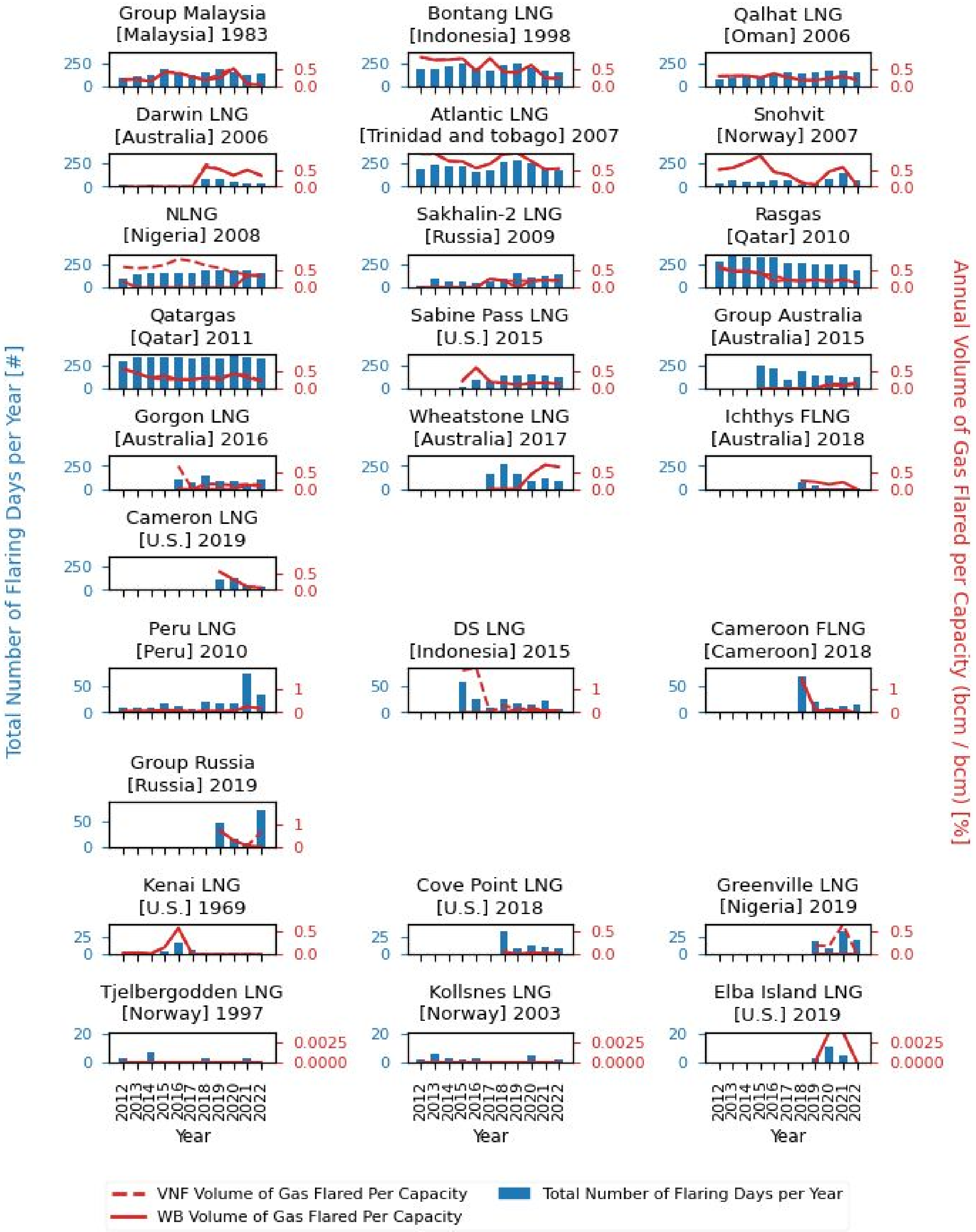

Figure S4. Annual number of flaring days per facility and comparison of the yearly volumes of gas flared per capacity provided by the World Bank (WB) and VIIRS Night Fire (VNF)

datasets for a selection of facilities. When dashed lines corresponding to the VNF estimates do not appear, it is because they are hidden by the solid line of the WB estimates (in that case, VNF and WB estimates are the same). The name of each facility is followed by its country (in parentheses) and the year it opened. The facilities are ordered by flaring activity; scales may vary between graphs but facilities with similar orders of magnitude are grouped together.

Table S6. Difference in volumes of gas flared (billion m^3) by each facility each year provided by VNF and WB

Facilities	2012	2013	2014	2015	2016	2017	2018	2019	2020	2021	2022	Total over 2012-2022
**AB LNG**	5E-02	6E-02	1E-01	1E-01	8E-02	8E-02	4E-02	6E-02	5E-02	1E-02	0E+00	7E-01
**ADNOC LNG**	8E-04	8E-04	7E-04	7E-04	-5E-03	0E+00	2E-03	0E+00	0E+00	0E+00	0E+00	3E-04
**Angola LNG**	1E-02	3E-02	2E-02	8E-03	-2E-02	0E+00	-7E-04	0E+00	0E+00	0E+00	0E+00	5E-02
**AP LNG, Gladstone, QC LNG**									1E-02	1E-02	1E-02	4E-02
**Atlantic LNG**	-2E-03	-2E-03	-1E-03	-1E-03	5E-04	0E+00	-3E-03	0E+00	0E+00	0E+00	0E+00	-8E-03
**Bahia Blanca FLNG**								5E-03	0E+00	1E-02	3E-03	2E-02
**Bontang LNG**	9E-04	1E-03	6E-04	1E-03	-5E-03	0E+00	9E-04	0E+00	0E+00	0E+00	0E+00	-4E-04
**Brunei LNG**	2E-03	6E-04	1E-03	-6E-04	4E-03	2E-02	5E-03	5E-03	3E-02	1E-02	1E-03	8E-02
**Calcasieu Pass**											0E+00	0E+00
**Cameron LNG**								0E+00	0E+00	0E+00	0E+00	0E+00
**Cameroon FLNG**							-3E-03	0E+00	0E+00	0E+00		-3E-03
**Corpus LNG**								0E+00	0E+00	0E+00	0E+00	0E+00
**Cove Point LNG**							-1E-04		0E+00	0E+00	0E+00	-1E-04
**CS FLNG**											1E-02	1E-02
**Damietta LNG**	0E+00	0E+00	0E+00	0E+00	1E-04	0E+00	5E-04	0E+00	0E+00	0E+00	0E+00	7E-04
**Darwin LNG**							3E-03	0E+00	0E+00	0E+00	5E-04	3E-03
**DS LNG**								0E+00	2E-03	0E+00	0E+00	2E-03
**Elba Island LNG**									0E+00	0E+00		0E+00
**ELNG**	-2E-04	-1E-04	-3E-04	-1E-04	3E-04	0E+00	-2E-03	0E+00	0E+00	0E+00	0E+00	-2E-03
**Gorgon LNG**										4E-03	6E-03	1E-02
**Greenville LNG**												0E+00
**Kenai LNG**	0E+00	0E+00	0E+00	0E+00	-1E-04							-1E-04
**Marsa LNG**											0E+00	0E+00
**MLNG, DM LNG, SM LNG, TM LNG**	1E-03	4E-04	5E-04	2E-03	1E-02	0E+00	0E+00	5E-02	0E+00	0E+00	0E+00	7E-02
**NLNG**	9E-02									5E-03	0E+00	1E-01
**North West Shelf**	-3E-03	-6E-03	-2E-03	-3E-03	9E-03	0E+00	3E-03	0E+00	0E+00	0E+00	-2E-03	-4E-03
**PE LNG**	1E-03	2E-03	2E-03	2E-03	-5E-04	0E+00	-1E-03	0E+00	0E+00	9E-03	0E+00	1E-02

**Peru LNG**	0E+00	0E+00	0E+00	-1E-04	-9E-04	0E+00	-1E-04	0E+00	0E+00	0E+00	0E+00	-1E-03
**Pluto LNG**	-3E-03	-2E-03	-4E-04	-6E-04	9E-03	0E+00	-9E-04	0E+00	0E+00	0E+00	0E+00	2E-03
**PNG LNG**		0E+00	-9E-04	-1E-04	4E-03	0E+00	0E+00	1E-04	0E+00	0E+00	0E+00	3E-03
**Prelude FLNG**								0E+00	0E+00	0E+00	0E+00	0E+00
**Qalhat LNG**	0E+00	0E+00	0E+00	0E+00	-4E-03	0E+00	-2E-04	0E+00	0E+00	0E+00	0E+00	-4E-03
**Qatargas**	-4E-03	-3E-03	-2E-03	-5E-02	6E-03	0E+00	3E-04	3E-02	0E+00	3E-02	0E+00	1E-02
**Rasgas**	-1E-02	-1E-02	-1E-02	-9E-03	6E-02	0E+00	-5E-03	0E+00	0E+00	0E+00	0E+00	2E-02
**Sabine Pass LNG**				4E-03	-1E-03	0E+00	-1E-04	0E+00	0E+00	0E+00	0E+00	2E-03
**Sakhalin-2 LNG**						0E+00	-6E-04		0E+00	0E+00	0E+00	-6E-04
**Skikda**								0E+00	0E+00	0E+00	3E-02	3E-02
**Tangguh LNG**										0E+00	0E+00	0E+00
**Vysotsk LNG, PO LNG**								0E+00	0E+00	0E+00		0E+00
**Wheatstone LNG**									0E+00	0E+00	0E+00	0E+00
**Yamal LNG**						4E-02	3E-03	4E-02	1E-03	0E+00	0E+00	9E-02
**Yemen LNG**	-2E-03	-7E-04	-1E-03	-1E-03	2E-03	0E+00	1E-04	0E+00	0E+00		0E+00	-2E-03

Table S7. Percentage difference in volumes of gas flared by each facility each year provided by VNF and WB

**Facilities**	**2012**	**2013**	**2014**	**2015**	**2016**	**2017**	**2018**	**2019**	**2020**	**2021**	**2022**	**Average**
**AB LNG**	8.7	9.5	12.8	17.8	12.4	21.2	9.4	14.1	9.3	2.4	0	10.7
**ADNOC LNG**	0.6	0.5	0.5	0.5	-3.5	0	1.1	0	0	0	0	0
**Angola LNG**	14	14.8	18.8	15.4	-5.2	0	-2.3	0	0	0	0	5.1
**AP LNG, Gladstone, QC LNG**									33.6	36.8	30.3	33.5
**Atlantic LNG**	-1.4	-1.4	-1.4	-1.4	0.8	0	-2.4	0	0	0	0	-0.7
**Bahia Blanca FLNG**								24.7	0	39.9	19.3	21
**Bontang LNG**	0.5	0.7	0.4	0.6	-5.5	0	1	0	0	0	0	-0.2
**Brunei LNG**	2.7	1.3	1.2	-0.6	5.8	23.7	11.4	9.7	95.9	71.8	8.9	21.1
**Calcasieu Pass**											0	0
**Cameron LNG**								0	0	0	0	0
**Cameroon FLNG**							-8.3	1	0.1	0.1		-1.8
**Corpus LNG**								0	0	0	0	0

**Cove Point LNG**							-3		0.2	0.2	-0.5	-0.8
**CS FLNG**											3	3
**Damietta LNG**	0	0.1	0	-0.2	1.6	0	7.2	0.1	0	0	0	0.8
**Darwin LNG**							10.2	0	0	0	3.7	2.8
**DS LNG**								0.9	54.1	0	0.3	13.8
**Elba Island LNG**									0.2	-0.2		0
**ELNG**	-0.2	-0.2	-0.4	-0.3	0.5	0	-5.9	0	0	0	0	-0.6
**Gorgon LNG**										15.5	33.9	24.7
**Greenville LNG**												
**Kenai LNG**	-0.8	-1.3	-0.3	-0.3	-0.6							-0.7
**Marsa LNG**											0	0
**MLNG, DM LNG, SM LNG, TM LNG**	1.3	0.5	0.8	0.9	6.7	0	0	34.6	0	0	0	4.1
**NLNG**	69.7									6.3	0	25.3
**North West Shelf**	-3	-3	-3.4	-3.3	18	0	5.3	0	0	0	-9.5	0.1
**PE LNG**	5.8	5.1	5.7	5.6	-1.2	0	-6.4	0.1	0	94.2	0	9.9
**Peru LNG**	-1.8	-1.8	-1.4	-1.6	-31.1	-0.1	-3.9	1.2	0.1	0	0	-3.7
**Pluto LNG**	-1.2	-1.2	-1.3	-1.2	36.1	0	-7.7	0	0	0	0	2.1
**PNG LNG**		-0.3	-0.4	-0.6	19	0	0	0.7	0.1	0	0	1.8
**Prelude FLNG**								0	0	0	0	0
**Qalhat LNG**	0.1	0	0	0.1	-11.5	0	-1	0	0	0	0	-1.1
**Qatargas**	-1.5	-1.5	-1.6	-44.2	5.4	0	0.2	22.2	0	19.5	0	-0.1
**Rasgas**	-5.8	-6.2	-6.5	-6.1	49.5	0	-8.2	0	0	0	0	1.5
**Sabine Pass LNG**				14.7	-2.1	0	-0.3	0	0	0	0	1.5
**Sakhalin-2 LNG**						0	-3.2		0	0	0	-0.7
**Skikda**								0	0	0	28	7
**Tangguh LNG**										0	0	0
**Vysotsk LNG, PO LNG**								0.2	-0.2	1.5		0.5
**Wheatstone LNG**									0	0	0	0
**Yamal LNG**						28	1.6	40.8	1.7	0	0	12
**Yemen LNG**	-1.3	-1.3	-1.3	-1.3	3.5	0	0.3	0	0		0	-0.1

S3. Analyses by case

S3.1. Case 1: Start-Up Conditions

S3.1.1. Number of Flaring Days Detected Every Year

Tables S8 provides descriptive information on the number of flaring days detected every year for start-up conditions considered between 1 and 4 years.

S3.1.2. Number of Consecutive Days with Flaring

Table S9 provides descriptive information on the number of consecutive days with flaring.

S3.1.3. Volume of Gas Flared Every Year

Tables S10 and S12 provide information on the yearly volumes of gas flared per capacity of the facilities for all facilities considered under case 1 (i.e., all facilities that opened in 2012 or after). In Table S12, data is sometimes missing for the first year(s) of operation, due to missing data from the VNF dataset.

We also compared volumes of gas flared per capacity for all facilities that opened since 2012 (Tables S10) vs. all facilities that opened since 2016 (Table S11), to analyze whether potential changes in technology/operation (based on lessons learned from previously opened facilities) could impact the number of flaring days and flaring events, and the amount of gas flared. There was no clear difference between those.

Table S8. Descriptive statistics of the number of flaring days detected per year for start-up conditions lasting between 1 and 4 years. The numbers in brackets correspond to the 95% confidence interval (i.e, 2.5% - 97.5%).

	**Number of years considered as start-up**							
	**1**		**2**		**3**		**4**	
	**Start-up**	**Normal**	**Start-up**	**Normal**	**Start-up**	**Normal**	**Start-up**	**Normal**
**mean**	111 (73, 155)	108 (94, 125)	119 (87, 149)	104 (88, 122)	109 (86, 131)	108 (90, 129)	107 (87, 127)	112 (90, 135)
**median**	86 (43, 111)	95 (74, 112)	96 (60, 151)	93 (61, 106)	90 (58, 127)	97 (70, 112)	93 (60, 111)	99 (66, 122)
**std**	94 (68, 118)	83 (73, 94)	97 (82, 110)	78 (67, 90)	92 (78, 105)	78 (66, 92)	88 (76, 100)	80 (64, 95)
**min**	3.0 (3.0, 3.0)	5.0 (5.0, 5.0)	3.0 (3.0, 3.0)	5.0 (5.0, 5.0)	3.0 (3.0, 3.0)	7.0 (7.0, 7.0)	3.0 (3.0, 3.0)	7.0 (7.0, 7.0)
**max**	296 (167, 296)	300 (283, 300)	297 (271, 297)	300 (263, 300)	297 (262, 297)	300 (263, 300)	297 (273, 297)	300 (239, 300)
**5%**	4.0 (3.0, 19)	9.0 (7.0, 11)	7.4 (3.0, 16)	10 (6.1, 12)	6.5 (3.0, 9.8)	12 (8.0, 18)	7.6 (4.0, 10)	15 (7.0, 22)
**95%**	290 (167, 296)	283 (209, 293)	293 (250, 297)	265 (203, 290)	291 (227, 296)	279 (200, 290)	280 (225, 296)	284 (211, 297)

Table S9. Descriptive statistics of the number of consecutive days with flaring for start-up conditions lasting between 1 and 4 years. The numbers in brackets correspond to the 95% confidence interval (i.e, 2.5% - 97.5%).

	**Number of years considered as start-up**							
	**1**		**2**		**3**		**4**	
	**Start-up**	**Normal**	**Start-up**	**Normal**	**Start-up**	**Normal**	**Start-up**	**Normal**
**mean**	3.8 (3.4, 4.4)	2.5 (2.4, 2.7)	3.6 (3.2, 3.9)	2.4 (2.2, 2.5)	3.2 (3.0, 3.4)	2.3 (2.2, 2.5)	3.0 (2.8, 3.2)	2.4 (2.2, 2.6)
**median**	2.0 (1.0, 1.0)	1.0 (1.0, 1.0)	2.0 (1.0, 1.0)	1.0 (1.0, 1.0)	1.0 (1.0, 1.0)	1.0 (1.0, 1.0)	1.0 (1.0, 1.0)	1.0 (1.0, 1.0)
**std**	6.7 (5.1, 9.2)	4.0 (3.4, 4.9)	6.0 (4.9, 7.4)	3.6 (2.9, 4.7)	5.4 (4.5, 6.6)	3.6 (2.8, 5.0)	4.9 (4.1, 5.8)	3.8 (2.7, 5.6)
**min**	1.0 (1.0, 1.0)	1.0 (1.0, 1.0)	1.0 (1.0, 1.0)	1.0 (1.0, 1.0)	1.0 (1.0, 1.0)	1.0 (1.0, 1.0)	1.0 (1.0, 1.0)	1.0 (1.0, 1.0)
**max**	94 (36, 94)	98 (41, 98)	94 (42. 94)	98 (32, 98)	94 (43, 94)	98 (28, 98)	94 (43, 94)	98 (24, 98)
**5%**	1.0 (1.0, 1.0)	1.0 (1.0, 1.0)	1.0 (1.0, 1.0)	1.0 (1.0, 1.0)	1.0 (1.0, 1.0)	1.0 (1.0, 1.0)	1.0 (1.0, 1.0)	1.0 (1.0, 1.0)
**95%**	14 (10, 14)	8.0 (7.0, 8.0)	13 (10, 15)	7.0 (6.0, 6.0)	11 (9.0, 12)	7.0 (6.0, 6.0)	10 (8.0, 11)	7.0 (6.0, 6.0)

Table S10. Descriptive statistics of the yearly volumes of gas flared per capacity (bcm/bcm [%]) for start-up conditions lasting between 1 and 4 years. The numbers in brackets correspond to the 95% confidence interval (i.e, 2.5% - 97.5%).

	**Number of years considered as start-up**							
	**1**		**2**		**3**		**4**	
	**Start-up**	**Normal**	**Start-up**	**Normal**	**Start-up**	**Normal**	**Start-up**	**Normal**
**mean**	2.4 (0.97, 4.6)	0.71 (0.50, 0.95)	1.9 (1.0, 3.2)	0.62 (0.43, 0.92)	1.4 (0.80, 2.3)	0.65 (0.44, 1.0)	1.2 (0.72, 2.0)	0.70 (0.42, 1.1)
**median**	0.71 (0.19, 1.8)	0.28 (0.17, 0.44)	0.70 (0.25, 1.8)	0.25 (0.16, 0.43)	0.57 (0.19, 0.86)	0.27 (0.16, 0.44)	0.37 (0.19, 0.65)	0.27 (0.15, 0.44)
**std**	3.5 (1.2, 5.3)	1.1 (0.71, 1.6)	2.8 (1.3, 4.3)	1.0 (0.56, 1.6)	2.4 (1.1, 3.8)	1.1 (0.51, 1.7)	2.1 (0.99, 3.3)	1.2 (0.58, 1.9)
**min**	0.041 (0.041, 0.041)	0.0038 (0.0038, 0.0038)	0.0039 (0.0039, 0.0039)	0.0038 (0.0038, 0.0038)	0.0038 (0.0038, 0.0038)	0.016 (0.016, 0.016)	0.0038 (0.0038, 0.0038)	0.016 (0.016, 0.016)
**max**	13 (1.8, 13)	6.9 (2.4, 6.9)	13 (2.7, 13)	6.9 (2.3, 6.9)	13 (2.6, 13)	6.9 (2.3, 6.9)	13 (2.8, 13)	6.9 (1.7, 6.9)
**5%**	0.053 (0.041, 0.11)	0.036 (0.0048, 0.082)	0.047 (0.0039, 0.11)	0.038 (0.01, 0.082)	0.035 (0.0038, 0.059)	0.069 (0.016, 0.093)	0.032 (0.0038, 0.067)	0.088 (0.016, 0.099)
**95%**	9.8 (2.7, 13)	2.5 (2.2, 3.8)	7.2 (2.7, 13)	2.3 (1.2, 3.3)	4.7 (2.1, 13)	2.3 (1.1, 3.8)	4.2 (2.3, 9.6)	2.5 (1.1, 6.3)

u

Table S11. Descriptive statistics of the yearly volumes of gas flared per capacity (bcm/bcm [%]) for start-up conditions lasting between 1 and 4 years, for all facilities that opened since 2016. The numbers in brackets correspond to the 95% confidence interval (i.e, 2.5% - 97.5%).

	**Number of years considered as start-up**							
	**1**		**2**		**3**		**4**	
	**Start up**	**Normal**	**Start up**	**Normal**	**Start up**	**Normal**	**Start up**	**Normal**
**mean**	2.8 (0.60, 5.6)	0.42 (0.26, 0.62)	1.8 (0.58, 3.8)	0.41 (0.24, 0.66)	1.3 (0.50, 2.6)	0.40 (0.23, 0.72)	1.1 (0.50, 2.1)	0.33 (0.16, 0.55)
**median**	0.70 (0.17, 2.8)	0.16 (0.10, 0.31)	0.42 (0.18, 1.0)	0.14 (0.09, 0.40)	0.24 (0.15, 0.64)	0.14 (0.10, 0.44)	0.19 (0.14, 0.53)	0.14 (0.09, 0.66)
**std**	4.1 (0.78, 5.7)	0.57 (0.27, 0.78)	3.3 (0.56, 5.1)	0.57 (0.24, 0.82)	2.8 (0.62, 4.34)	0.52 (0.23, 0.88)	2.4 (0.66, 4.0)	0.29 (0.20, 0.34)
**min**	0.058 (0.058, 0.058)	0.0038 (0.0038, 0.0038)	0.0039 (0.0039, 0.0039)	0.0038 (0.0038, 0.0038)	0.0038 (0.0038, 0.0038)	0.016 (0.016, 0.016)	0.0038 (0.0038, 0.0038)	0.0156 (0.0156, 0.0156)
**max**	123 (1.4, 13)	2.3 (0.72, 2.3)	13 (1.4, 13)	2.3 (0.66, 2.3)	13 (1.4, 13)	2.3 (0.65, 2.3)	13 (1.7, 13)	0.78 (0.30, 0.78)
**5%**	0.094 (0.058, 0.16)	0.013 (0.0038, 0.039)	0.05 (0.0039, 0.087)	0.019 (0.0038, 0.047)	0.015 (0.0038, 0.058)	0.023 (0.016, 0.063)	0.02 (0.0038, 0.04)	0.047 (0.016, 0.094)
**95%**	11 (1.1, 13)	1.8 (0.66, 2.3)	9.1 (1.4, 13)	1.7 (0.65, 2.3)	6.5 (1.3, 13)	1.0 (0.65, 2.3)	3.9 (1.4, 13)	0.75 (0.32, 0.78)

Table S12. Volumes of gas flared per capacity (bcm/bcm [%]) for each year for facilities that opened after 2012 (inclusive)

**Facilities**	**Year since starting**										
	**1**	**2**	**3**	**4**	**5**	**6**	**7**	**8**	**9**	**10**	**11**
Angola LNG	1.795	3.981	1.799	0.975	6.848	1.094	0.560	0.829	0.878	1.208	0.936
AP LNG, Gladstone, QC LNG						0.138	0.119	0.163			
Calcasieu Pass	0.139										
Cameron LNG	0.532	0.308	0.098	0.070							
Cameroon FLNG	1.375	0.092	0.082	0.082							
Corpus LNG		0.185	0.168	0.142	0.094						
Cove Point LNG	0.058		0.035	0.024	0.016						
CS FLNG	12.806										
DS LNG	1.820	1.952		0.300	0.134	0.185	0.086	0.060			
Elba Island LNG		0.004	0.004								
Gorgon LNG	0.679		0.157	0.145	0.114	0.143	0.120				
Greenville LNG	0.190	0.179	0.643								
Pluto LNG	4.684	2.456	0.646	1.049	0.480	0.417	0.240	0.435	0.418	0.455	0.291
PNG LNG	0.041	2.706	0.276	0.253	0.150	0.294	0.098	0.095	0.164	0.157	
Prelude FLNG	8.486	1.710	2.248	2.304							
Sabine Pass LNG	0.246	0.601	0.195	0.158	0.101	0.152	0.168	0.131			
Skikda							2.254	3.245	2.478	2.310	
Vysotsk LNG, PO LNG	0.712	0.280	0.040	0.646							
Wheatstone LNG				0.439	0.714	0.655					
Yamal LNG	2.761	1.035	0.629	0.437	0.779	0.319					

S3.1.4. Case 2: Regular operating conditions – No start-up

Figure S5 provides details on the yearly numbers of flaring days, number of consecutive days with flaring, and volumes of gas flared per capacity for the facilities operating under regular conditions. Tables S13 and S14 provide descriptive statistics and estimates of volumes of gas flared per capacity.

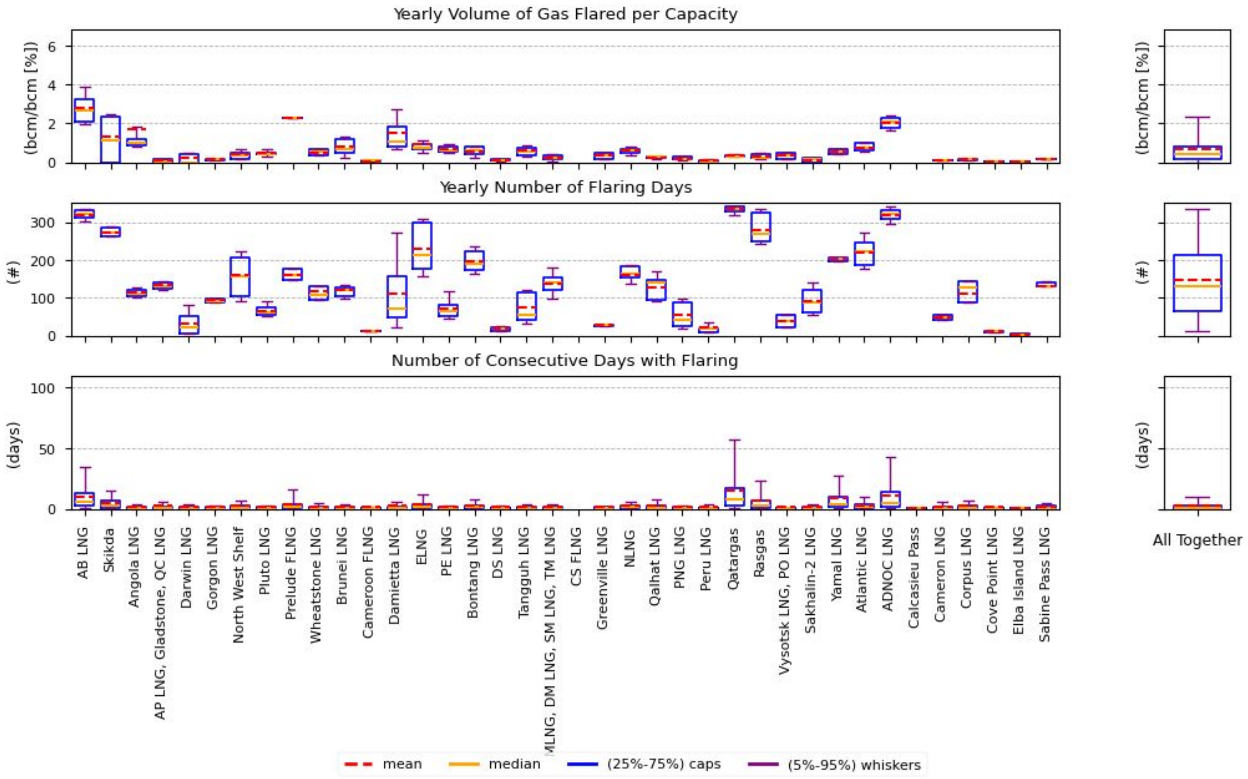

Figure S5. Analysis of yearly numbers of flaring days, number of consecutive days with flaring, and volumes of gas flared per capacity for facilities opened for at least 3 years.

Table S13. Descriptive statistics of yearly volume of gas flared per capacity (bcm/bcm [%]), number of flaring days per year (#), and number of consecutive days with flaring (days) for years 3+ after start-up of the facility. The numbers in brackets correspond to the 95% confidence interval (i.e, 2.5% - 97.5%).

	**Yearly volumes of gas flared per capacity (bcm/bcm [%])**	**Number of flares detected per year (#)**	**Number of consecutive days with flaring (days)**
**mean**	0.73 (0.64, 0.85)	148 (137, 159)	3.1 (3.0, 3.3)
**median**	0.45 (0.39, 0.53)	130 (118, 144)	1.0 (1.0, 1.0)
**std**	0.86 (0.70, 1.1)	101 (95, 107)	5.9 (5.5, 6.4)
**min**	0.0038 (0.0038, 0.0038)	4.0 (4.0, 4.0)	1.0 (1.0, 1.0)
**max**	6.9 (3.3, 6.9)	353 (341, 353)	110 (87, 110)
**5%**	0.06 (0.04, 0.086)	10 (6.5, 14)	1.0 (1.0, 1.0)
**95%**	2.4 (2.2, 3.0)	334 (322, 335)	10 (9.0, 9.0)

Table S14. Volumes of gas flared per capacity (bcm/bcm [%]) for years 3+ after start-up

**Facilities**	**2012**	**2013**	**2014**	**2015**	**2016**	**2017**	**2018**	**2019**	**2020**	**2021**	**2022**
AB LNG	3.55	3.89	4.34	2.93	2.96	1.83	1.93	2.09	2.67	2.06	2.15
ADNOC LNG	1.61	2.09	1.93	1.64	1.70	1.79	2.34	2.26	2.37	2.23	2.41
Angola LNG			1.80	0.97	6.85	1.09	0.56	0.83	0.88	1.21	0.94
AP LNG, Gladstone, QC LNG									0.14	0.12	0.16
Atlantic LNG	0.97	0.97	0.75	0.73	0.55	0.68	0.95	0.99	0.75	0.51	0.53
Bontang LNG	0.85	0.77	0.78	0.81	0.43	0.81	0.41	0.40	0.62	0.24	0.22
Brunei LNG	1.13	0.70	1.20	1.30	1.03	1.40	0.55	0.67	0.41	0.24	0.19
Cameron LNG										0.10	0.07
Cameroon FLNG									0.08	0.08	
Corpus LNG									0.17	0.14	0.09
Cove Point LNG									0.04	0.02	0.02
Damietta LNG	3.90	2.02	2.72	1.54	0.88	0.66	0.75	1.59	0.69	0.91	1.05
Darwin LNG							0.66	0.51	0.33	0.49	0.34
DS LNG							0.30	0.13	0.19	0.09	0.06
Elba Island LNG										0.00	
ELNG	0.97	0.72	1.10	0.69	0.69	0.50	0.45	0.87	0.72	2.60	0.75
Gorgon LNG							0.16	0.14	0.11	0.14	0.12
Greenville LNG										0.64	
MLNG, DM LNG, SM LNG, TM LNG	0.18	0.20	0.14	0.41	0.40	0.27	0.18	0.36	0.52	0.06	0.04
NLNG	0.61	0.57	0.59	0.67	0.84	0.78	0.65	0.57	0.45	0.38	0.35
North West Shelf	0.64	1.13	0.43	0.46	0.32	0.55	0.34	0.17	0.15	0.11	0.15
PE LNG	0.64	0.94	0.80	0.80	1.07	0.82	0.57	0.63	0.50	0.25	0.52
Peru LNG	0.05	0.05	0.06	0.10	0.06	0.03	0.06	0.05	0.07	0.24	0.17
Pluto LNG			0.65	1.05	0.48	0.42	0.24	0.44	0.42	0.45	0.29
PNG LNG				0.28	0.25	0.15	0.29	0.10	0.10	0.16	0.16
Prelude FLNG										2.25	2.30
Qalhat LNG	0.29	0.30	0.30	0.25	0.34	0.26	0.18	0.17	0.22	0.29	0.19
Qatargas		0.44	0.30	0.26	0.27	0.26	0.32	0.32	0.43	0.39	0.21
Rasgas	0.57	0.47	0.47	0.41	0.36	0.22	0.18	0.23	0.18	0.24	0.14
Sabine Pass LNG						0.20	0.16	0.10	0.15	0.17	0.13
Sakhalin-2 LNG						0.26	0.19	0.20	0.19	0.22	0.20
Skikda								2.25	3.25	2.48	2.31
Tangguh LNG	1.43	0.89	0.76	0.34	0.51	0.65	0.58	0.27	0.48	0.34	0.13
Vysotsk LNG, PO LNG										0.04	0.65
Wheatstone LNG									0.44	0.71	0.66
Yamal LNG								0.63	0.44	0.78	0.32

S3.2. Case 3: Regular vs. Irregular Conditions

Figure S6 provides details on the yearly numbers of flaring days, number of consecutive days with flaring, and volumes of gas flared per capacity for the facilities with continuous vs. non-continuous status. Tables S15 and S16 provide descriptive statistics on the yearly volume of gas flared per capacity, number of flaring days per year, and number of consecutive days with flaring for facilities with an interrupted and a continuous operating status, respectively.

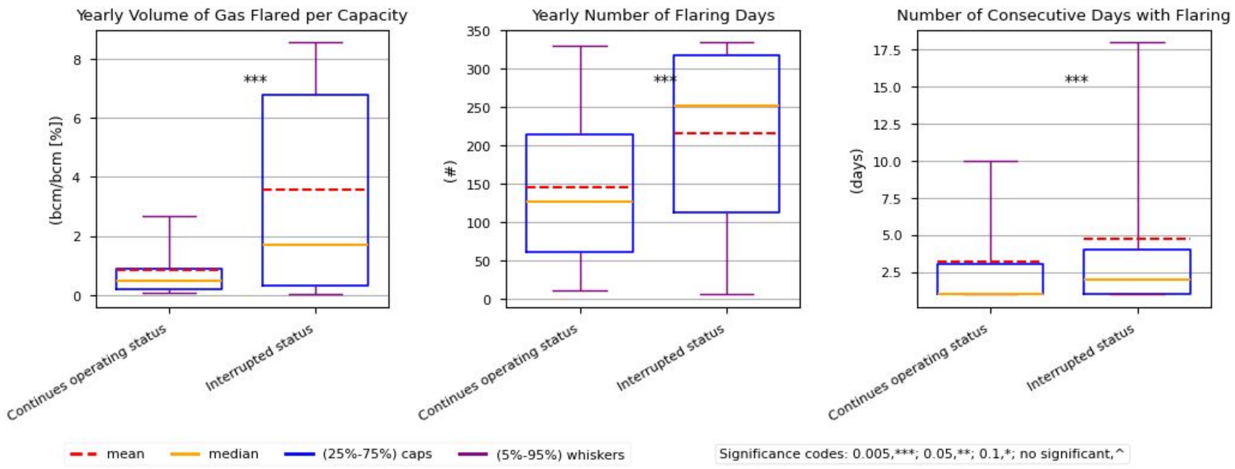

Figure S6. Analysis of yearly numbers of flaring days, number of consecutive days with flaring, and volumes of gas flared per capacity for facilities with a continuous status and interrupted status. The significance codes are based on t-tests.

Table S15. Descriptive statistics of yearly volume of gas flared per capacity (bcm/bcm [%]), number of flaring days per year (#), and number of consecutive days with flaring (days) for the 5 facilities with an interrupted operating status. The numbers in brackets correspond to the 95% confidence interval (i.e, 2.5% - 97.5%).

	**Yearly volumes of gas flared per capacity (bcm/bcm [%])**	**Number of flaring days detected per year (days)**	**Number of consecutive days with flaring (days)**
**mean**	3.6 (2.4, 4.7)	215 (170, 255)	4.8 (4.3, 5.2)
**median**	1.7 (0.39, 5.0)	251 (119, 303)	2.0 (2.0, 2.0)
**std**	3.5 (2.9, 4.0)	115 (93, 133)	9.1 (7.4, 11)
**min**	0.016 (0.016, 0.016)	1.0 (1.0, 1.0)	1.0 (1.0, 1.0)
**max**	11 (7.8, 11)	341 (323, 341)	122 (62, 122)
**5%**	0.034 (0.016, 0.14)	5.5 (1.0, 51)	1.0 (1.0, 1.0)
**95%**	9.1 (7.4, 11)	335 (323, 341)	18 (15, 20)

Table S16. Descriptive statistics of yearly volume of gas flared per capacity (bcm/bcm [%]), number of flaring days per year (#), and number of consecutive days with flaring (days) for the facilities with a continuous operating status. The numbers in brackets correspond to the 95% confidence interval (i.e, 2.5% - 97.5%).

	**Yearly volumes of gas flared per capacity (bcm/bcm [%])**	**Number of flaring days detected per year (days)**	**Number of consecutive days with flaring (days)**
**mean**	0.84 (0.72, 1.02)	145 (133, 155)	3.2 (3.1, 3.3)
**median**	0.47 (0.41, 0.55)	127 (112, 138)	1.0 (1.0, 1.0)
**std**	1.2 (0.85, 1.7)	101 (95, 106)	6.0 (5.5, 6.4)
**min**	0.0038 (0.0038, 0.0038)	3.0 (3.0, 3.0)	1.0 (1.0, 1.0)
**max**	13 (3.9, 13)	353 (341, 353)	110 (89, 110)
**5%**	0.059 (0.041, 0.088)	9.9 (7.0, 16)	1.0 (1.0, 1.0)
**95%**	2.7 (2.3, 3.5)	330 (322, 335)	10 (10, 10)

S3.3. Case 4: Offshore vs. Onshore

Figure S7 provides details on the yearly numbers of flaring days, number of consecutive days with flaring, and volumes of gas flared per capacity for onshore vs. offshore facilities. Tables S17 and S18 provide descriptive statistics on the yearly volume of gas flared per capacity, number of flaring days per year, and number of consecutive days with flaring for onshore and offshore facilities, respectively.

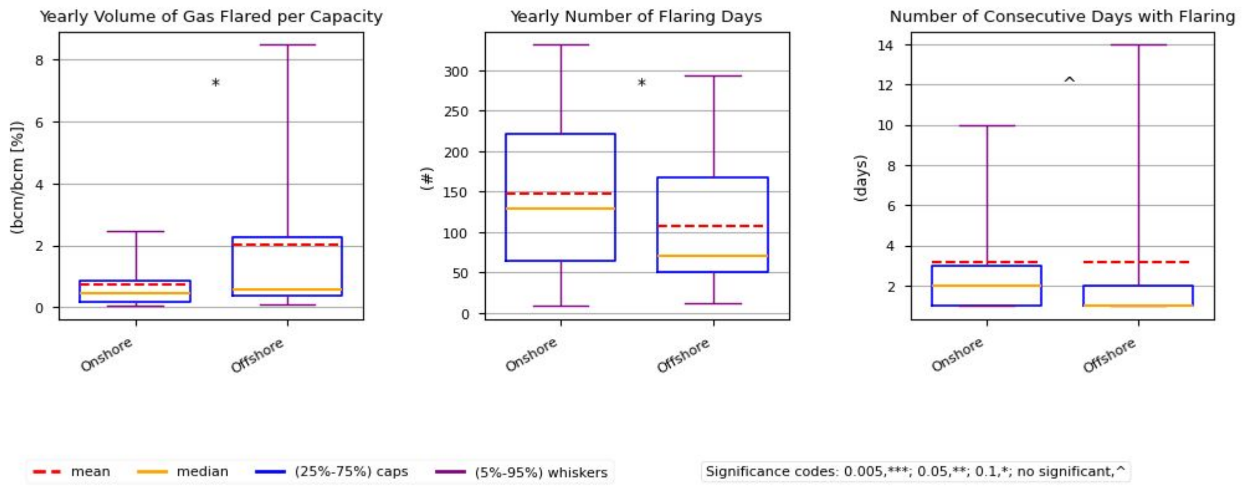

Figure S7. Analysis of yearly numbers of flaring days, number of consecutive days with flaring, and volumes of gas flared per capacity for onshore and offshore facilities. The significance codes are based on t-tests.

Table S17. Descriptive statistics of yearly volume of gas flared per capacity (bcm/bcm [%]), number of flaring days per year (#), and number of consecutive days with flaring (days) for the onshore facilities with a continuous operating status. The numbers in brackets correspond to the 95% confidence interval (i.e, 2.5% - 97.5%).

	Yearly volumes of gas flared per capacity (bcm/bcm [%])	Number of flares detected per year (days)	Number of consecutive days with flaring (days)
**mean**	0.75 (0.65, 0.86)	147 (136, 159)	3.2 (3.1, 3.3)
**median**	0.47 (0.39, 0.55)	130 (119, 145)	2.0 (1.0, 2.0)
**std**	0.89 (0.72, 1.1)	101 (95, 107)	6.0 (5.5, 6.5)
**min**	0.0038 (0.0038, 0.0038)	3.0 (3.0, 3.0)	1.0 (1.0, 1.0)
**max**	6.9 (3.7, 6.9)	353 (340, 353)	110 (88, 110)
**5%**	0.058 (0.04, 0.089)	9.0 (6.9, 16)	1.0 (1.0, 1.0)
**95%**	2.6 (2.1, 2.9)	332 (322, 337)	10 (10, 10)

Table S18. Descriptive statistics of yearly volume of gas flared per capacity (bcm/bcm [%]), number of flaring days per year (#), and number of consecutive days with flaring (days) for the offshore facilities with a continuous operating status. The numbers in brackets correspond to the 95% confidence interval (i.e, 2.5% - 97.5%).

	Yearly volumes of gas flared per capacity (bcm/bcm [%])	Number of flares detected per year (days)	Number of consecutive days with flaring (days)
**mean**	2.0 (0.94, 3.7)	107 (71, 147)	3.2 (2.7, 3.7)
**median**	0.56 (0.35, 1.7)	71 (50, 131)	1.0 (1.0, 1.0)
**std**	3.2 (1.1, 4.8)	89 (64, 111)	6.3 (4.5, 8.7)
**min**	0.082 (0.082, 0.082)	10 (10, 10)	1.0 (1.0, 1.0)
**max**	13 (2.3, 13)	296 (176, 296)	94 (32, 94)
**5%**	0.082 (0.082, 0.092)	12 (10, 14)	1.0 (1.0, 1.0)
**95%**	8.7 (2.3, 13)	293 (167, 296)	14 (8.0, 16)

S3.4. Probabilities of Flaring Events and Associated Volumes of Gas Flared per Life Stage

S3.4.1. Multiple Distributions Tested

We tested the following: 1) log-normal; 2) generalized extreme value (GEV); 3) Gumbal; 4) exponential; and 5) generalized Patero. We present here the results of this fitting exercise. The following figures display the q-q plots for the fitted distributions for a) yearly volume of gas flared per capacity during the start-up period (Figure S8); b) yearly number of flaring days during the start-up period (Figure S9); c) number of consecutive days with flaring during the start-up period (Figure S10); d) yearly volume of gas flared per capacity during regular operations (Figure S11); e) yearly number of flaring days during regular operations (Figure S12); and f) number of consecutive days with flaring during regular operations (Figure S13).

To further assess the goodness of fit of the different distributions, we calculated the Bayesian Information Criterion (BIC) as in equation (S4) to identify the best fitting models using the lowest BIC value. Table S19 summarizes the BIC values of each fitted model, with the lowest BIC value for each variable.

$$BIC = k\,ln(n) - 2\,ln(\hat{L}) \qquad \text{(S4)}$$

where k is the number of parameters estimated by the model, n is the number of observations, and $\hat{L}$ is the maximized value of the likelihood function of the model.

The log-normal distribution performs better than the other distributions, which is why it is used in the main manuscript.

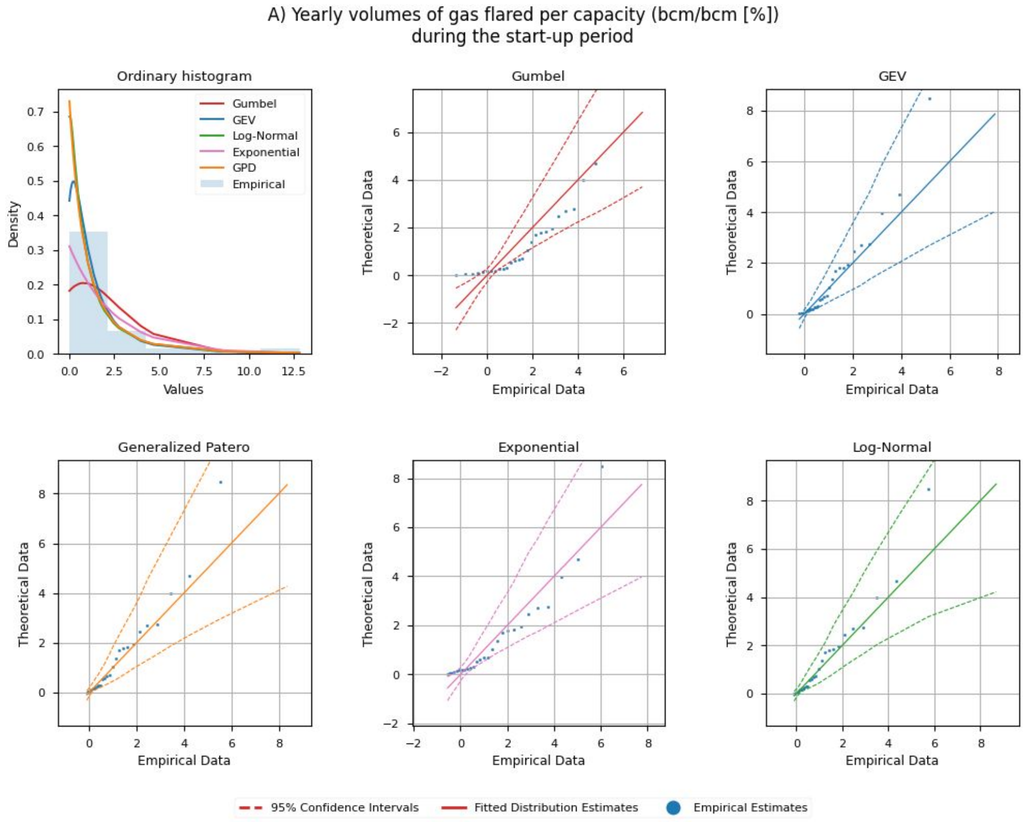

Figure S8. Q-Q plots of fitted Gumbel, GEV (generalized extreme value), Generalized Patero, Exponential and Log-Normal distributions of yearly volume of gas flared per capacity during the start-up period

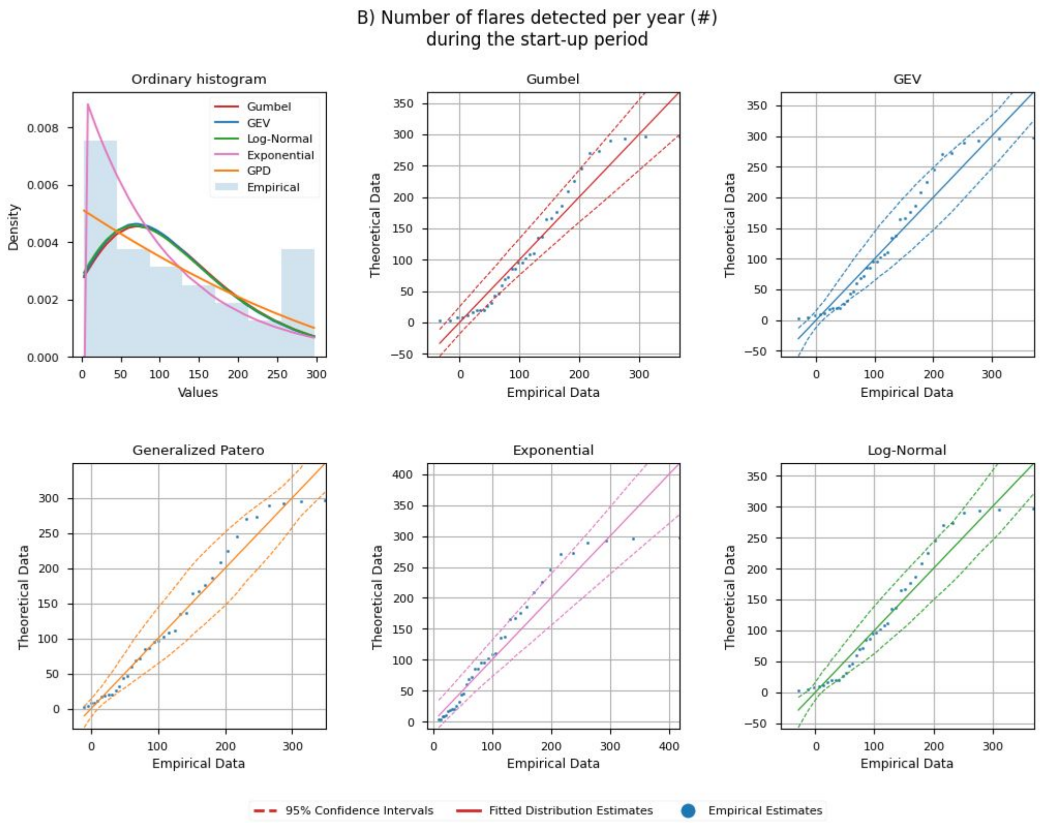

Figure S9. Q-Q plots of fitted Gumbel, GEV (generalized extreme value), Generalized Patero, Exponential and Log-Normal distributions of yearly number of flaring days during the start-up period

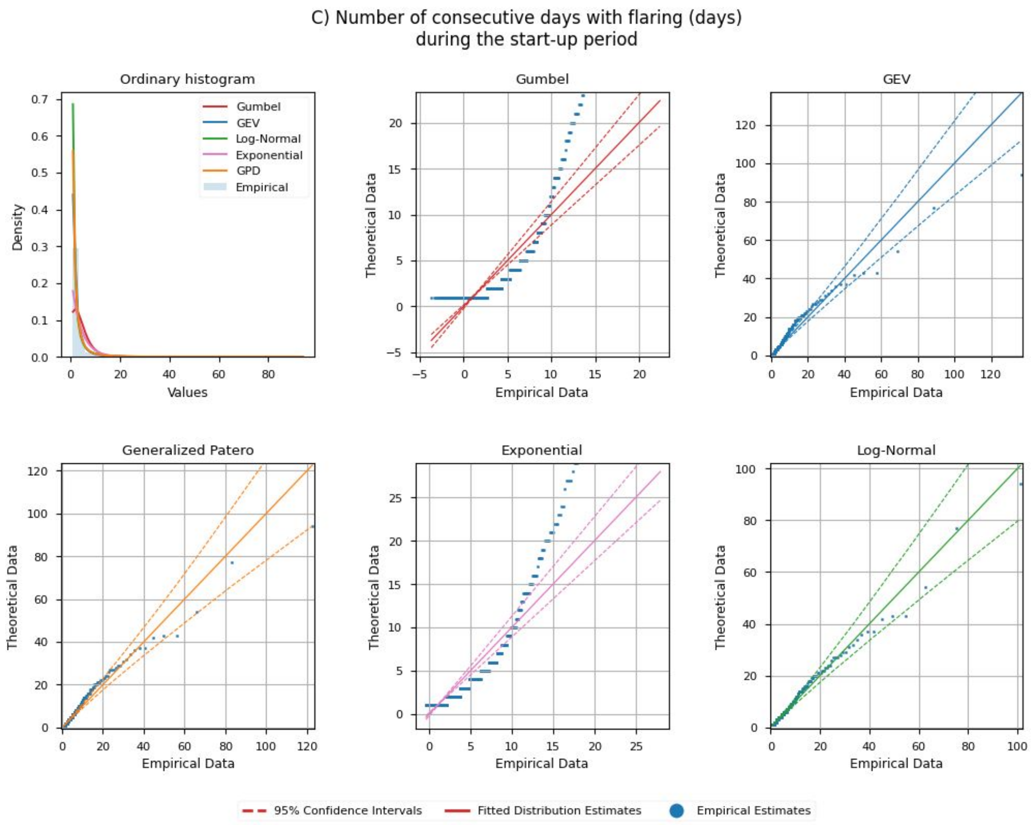

Figure S10. Q-Q plots of fitted Gumbel, GEV (generalized extreme value), Generalized Patero, Exponential and Log-Normal distributions of number of consecutive days with flaring during the start-up period

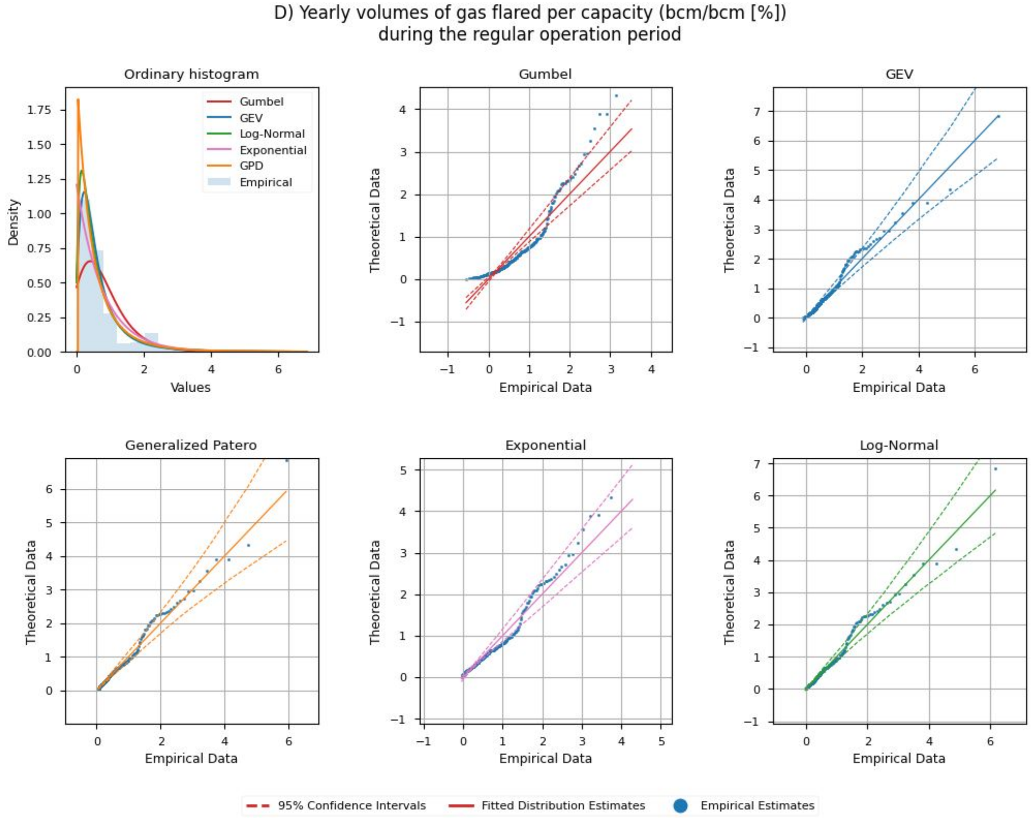

Figure S11. Q-Q plots of fitted Gumbel, GEV (generalized extreme value), Generalized Patero, Exponential and Log-Normal distributions of yearly volume of gas flared per capacity during regular operations

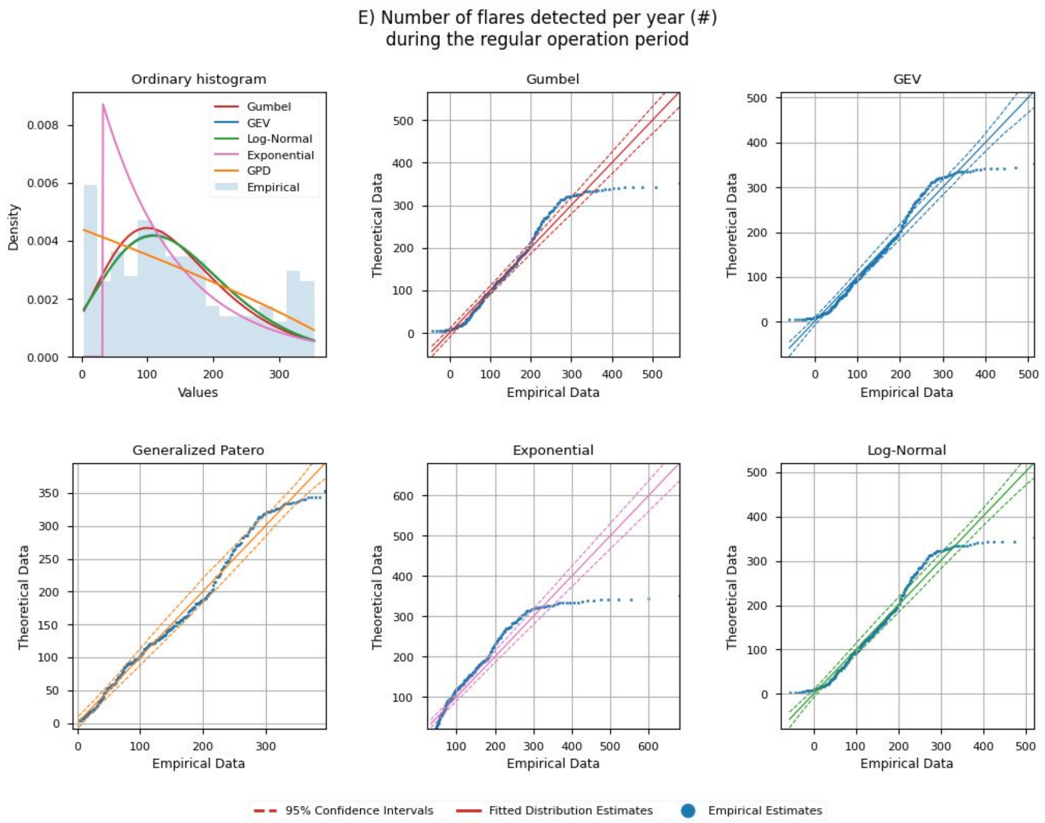

Figure S12. Q-Q plots of fitted Gumbel, GEV (generalized extreme value), Generalized Patero, Exponential and Log-Normal distributions of yearly number of flaring days during regular operations

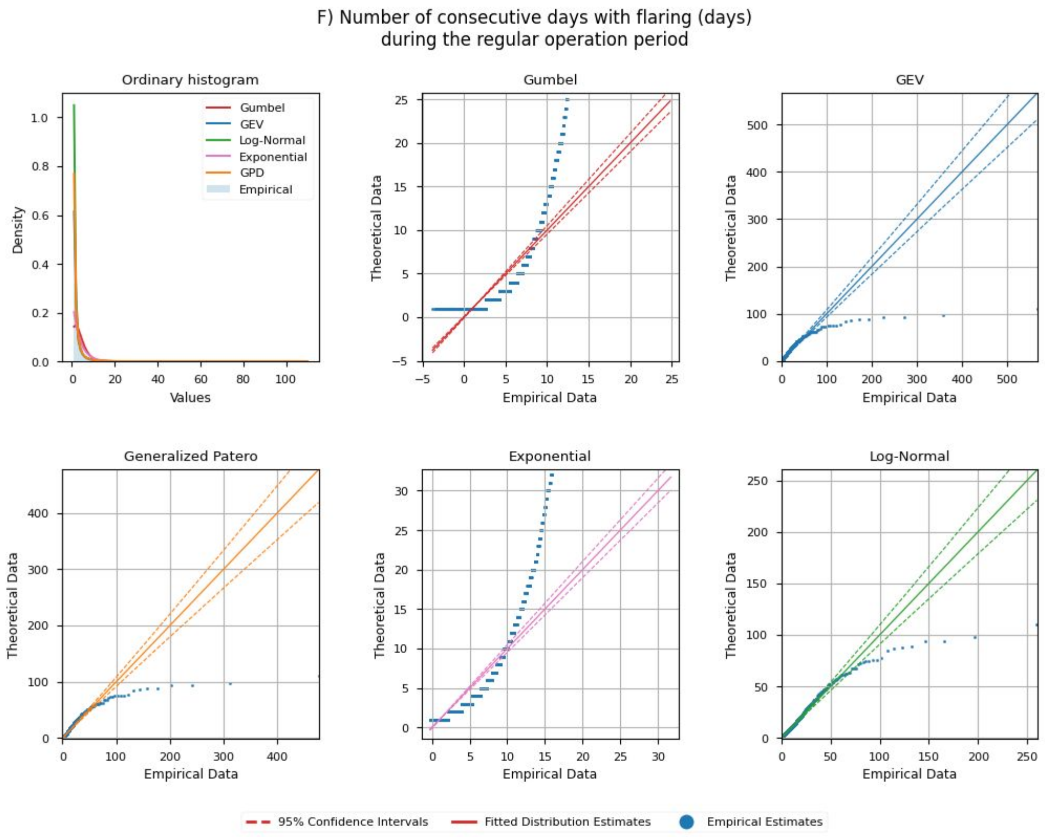

Figure S13. Q-Q plots of fitted Gumbel, GEV (generalized extreme value), Generalized Patero, Exponential and Log-Normal distributions of number of consecutive days with flaring during regular operations

Table S19. Bayesian Information Criterion (BIC) of each fitted model

		Gumbel	GEV (generalized extreme value)	Log-Normal	Exponential	Generalized Patero
**Start-up**	**Annual number of flaring days**	458	461	460	N/A	**453**
	**Annual volume of gas flared**	120	103	**99**	111	**99**
	**Number of consecutive days with flaring**	6,973	5,160	**4,698**	6,437	4,913
**Regular Operation**	**Annual number of flaring days**	3,837	3,844	3,843	NA	**3,767**
	**Annual volume of gas flared**	546	425	**405**	443	NA
	**Number of consecutive days with flaring**	62,111	41,613	**36,032**	56,955	39,190

S3.4.2. Log-Normal Distribution

Figures S14 and S15 show the q-q plots for the fitted log-normal distributions for the yearly numbers of flaring days, number of consecutive days with flaring and yearly volumes of gas flared per capacity for both the start-up and regular operation conditions. p-values for the two goodness-of-fit tests (i.e., one-sample Cramér-von Mises test and one-sample Kolmogorov-Smirnov test) are provided in Table S20.

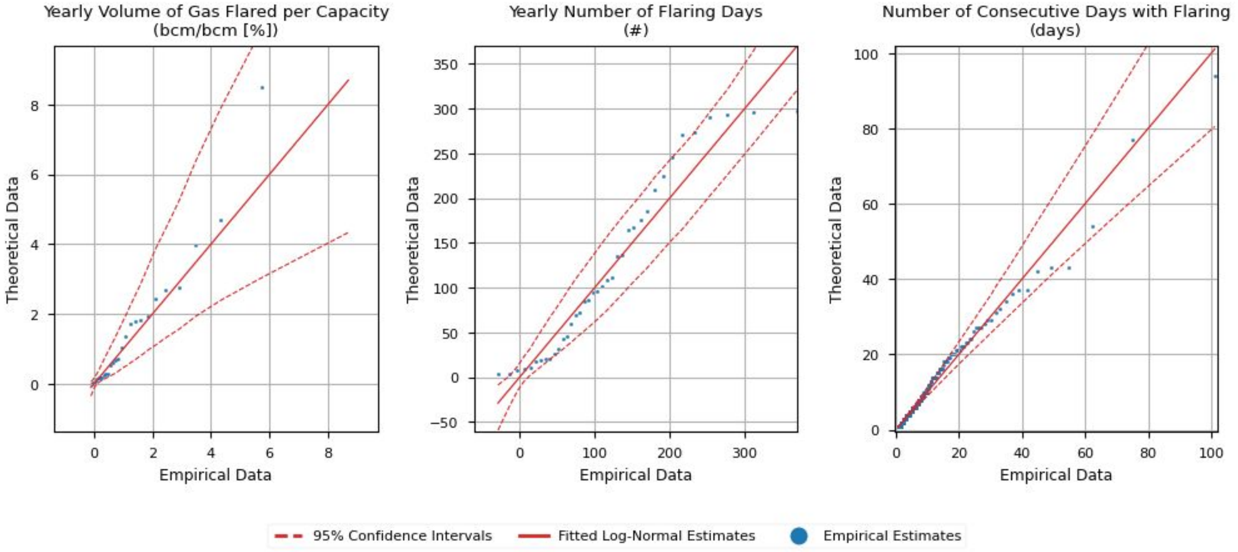

Figure S14. Q-Q plots of fitted log-normal distribution of yearly number of flaring days, number of consecutive days with flaring, and yearly volume of gas flared per capacity for the start-up period.

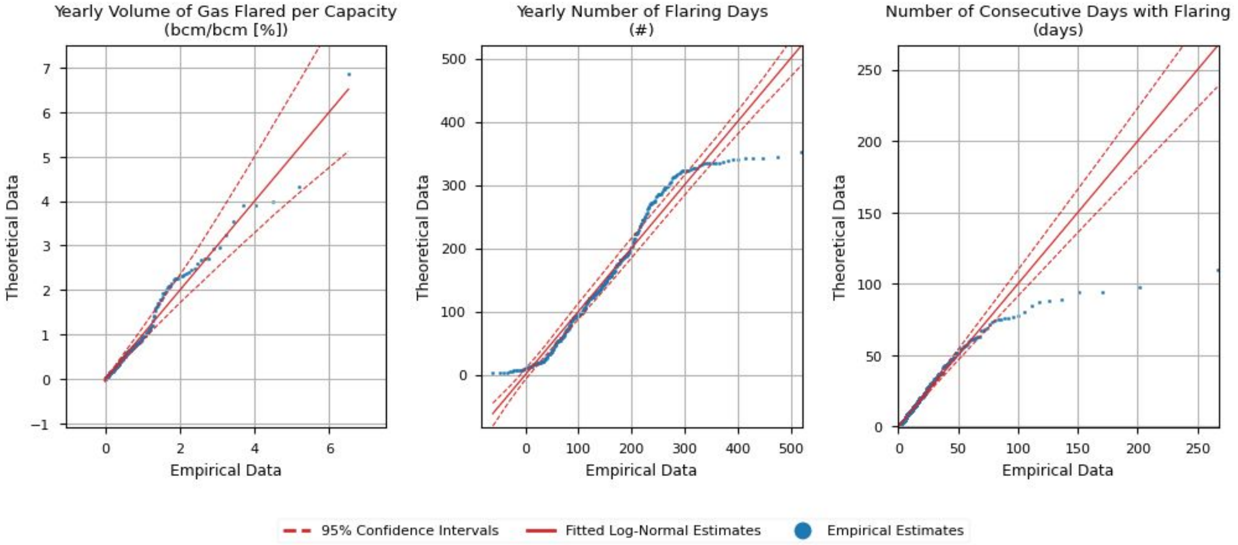

Figure S15. Q-Q plots of fitted log-normal distribution of yearly number of flaring days, number of consecutive days with flaring, and yearly volume of gas flared per capacity for the only regular operations periods.

Table S20. p-value for two goodness-of-fit tests (i.e., one-sample Cramér-von Mises test and one-sample Kolmogorov-Smirnov test) of yearly number of flaring days number of consecutive days with flaring, and yearly volume of gas flared per capacity for start-up and regular operation condition

	**one-sample Cramér-von Mises test**		**one-sample Kolmogorov-Smirnov test**	
	**Start-up**	**Regular Operations**	**Start-up**	**Regular Operations**
**Yearly Number of Flaring Days**	8.6E-01	7.3E-01	9.1E-01	7.8E-01
**Number of Consecutive Days with Flaring**	7.0E-01	2.3E-01	8.6E-01	1.9E-01
**Yearly Volume of Gas Flared per Capacity**	4.8E-10	3.8E-08	1.4E-93	0.0E+00

Table S21 displays the ratio of 1) annual number of flaring days and 2) volume of gas flared per capacity; during start-up compared to regular operation for various probabilities.

Table S21. Ratio of 1) annual number of flaring days and 2) volume of gas flared per capacity; during start-up compared to regular operation for various probabilities (1-exceedance probability).

**Probability [%]**	**Yearly volumes of gas flared per capacity (bcm/bcm [%])**	**Number of days with flaring per year (days)**
**90**	0.082	0.19
**80**	0.89	0.55
**70**	1.2	0.65
**60**	1.5	0.71
**50**	1.8	0.74
**40**	2.0	0.78
**30**	2.2	0.82
**20**	2.3	0.85
**10**	2.8	0.89

Analysis Including Kiyanly LNG

S3.5. Kiyanly LNG

Table S22. Descriptive statistics of yearly volume of gas flared per capacity (bcm/bcm [%]), number of flaring days per year (#), and number of consecutive days with flaring (days) of Kiyanly LNG. The numbers in brackets correspond to the 95% confidence interval (i.e, 2.5% - 97.5%).

	**Yearly volumes of gas flared per capacity (bcm/bcm [%])**	**Number of flares detected per year (days)**	**Number of consecutive days with flaring (days)**
**mean**	56 (32, 81)	259 (246, 270)	5.3 (4.7, 6.0)
**median**	39 (17, 96)	262 (235, 266)	3.0 (2.0, 3.0)
**std**	41 (33, 47)	20 (14, 25)	7.4 (6.0, 9.3)
**min**	15 (15, 15)	227 (227, 227)	1.0 (1.0, 1.0)
**max**	116 (51, 116)	287 (264, 287)	72 (35, 72)
**5%**	16 (15, 17)	23 (227, 238)	1.0 (1.0, 1.0)
**95%**	113 (73, 116)	287 (266, 287)	19 (15, 25)

S3.6. Case 2: Regular Operating Conditions – No Start-Up

In this section, we compare the descriptive statistics of all facilities excluding Kiyanly LNG (results provided in the main manuscript), all facilities including Kiyanly LNG, and Kiyanly LNG only.

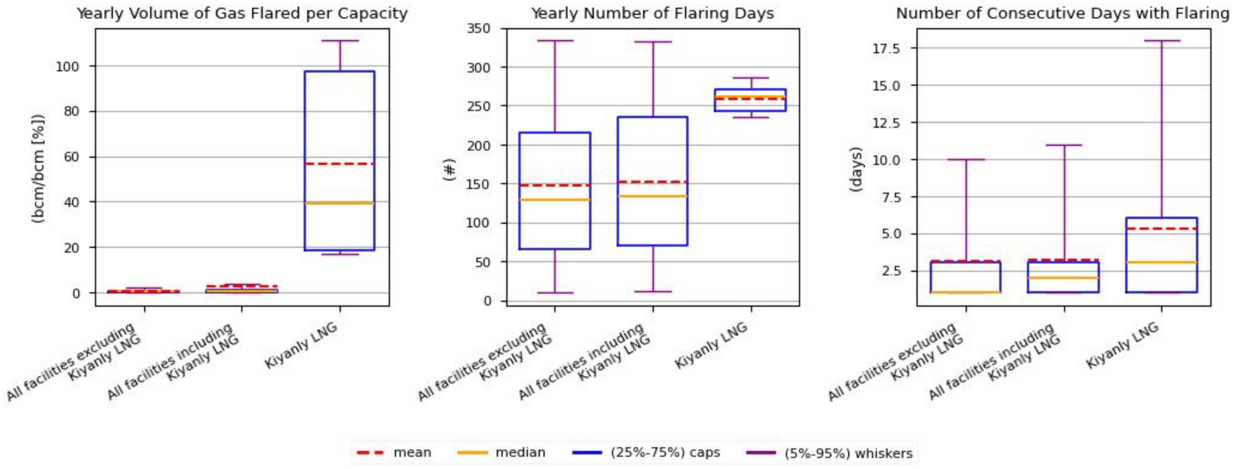

Figure S16. Analysis of yearly numbers of flaring days, number of consecutive days with flaring, and volumes of gas flared per capacity for only regular operation status facilities. The data distributions represent the primary analysis's facilities (All facilities excluding Kiyanly LNG – results provided in the main manuscript), primary analysis's facilities including Kiyanly LNG (All facilities excluding Kiyanly LNG), and Kiyanly LNG only.

Table S23. Descriptive statistics of yearly volume of gas flared per capacity (bcm/bcm [%]) for all facilities excluding Kiyanly LNG, for Kiyanly LNG and for all facilities including Kiyanly LNG. The numbers in brackets correspond to the 95% confidence interval (i.e, 2.5% - 97.5%).

	All facilities excluding Kiyanly LNG	All facilities including Kiyanly LNG	Kiyanly LNG
**mean**	0.73 (0.64, 0.85)	2.8 (1.5, 4.7)	56 (32, 81)
**median**	0.45 (0.39, 0.53)	0.48 (0.42, 0.57)	39 (17, 96)
**std**	0.86 (0.70, 1.1)	13 (7.1, 19)	41 (33, 47)
**min**	0.0038 (0.0038, 0.0038)	0.0038 (0.0038, 0.0038)	15 (15, 15)
**max**	6.9 (3.3, 6.9)	116 (27, 116)	116 (51, 116)
**5%**	0.06 (0.04, 0.086)	0.062 (0.047, 0.096)	16 (15, 17)
**95%**	2.4 (2.2, 2.9)	3.8 (2.5, 17)	113 (73, 116)

Table S24. Descriptive statistics of number of flaring days per year (#) for all facilities excluding Kiyanly LNG, for Kiyanly LNG and for all facilities including Kiyanly LNG. The numbers in brackets correspond to the 95% confidence interval (i.e, 2.5% - 97.5%).

	All facilities excluding Kiyanly LNG	All facilities including Kiyanly LNG	Kiyanly LNG
**mean**	148 (137, 159)	152 (140, 164)	259 (246, 270)
**median**	130 (118, 144)	133 (121, 148)	262 (235, 266)
**std**	101 (95, 107)	101 (95, 107)	20 (14, 25)
**min**	4.0 (4.0, 4.0)	4.0 (4.0, 4.0)	227 (227, 227)
**max**	353 (341, 353)	353 (341, 353)	287 (264, 287)
**5%**	10 (6.5, 14)	11 (7.5, 18)	23 (227, 238)
**95%**	334 (322, 335)	333 (323, 336)	287 (266, 287)

Table S25. Descriptive statistics of number of consecutive days with flaring (days) for all facilities excluding Kiyanly LNG, for Kiyanly LNG and for all facilities including Kiyanly LNG. The numbers in brackets correspond to the 95% confidence interval (i.e, 2.5% - 97.5%).

	All facilities excluding Kiyanly LNG	All facilities including Kiyanly LNG	Kiyanly LNG
**mean**	3.1 (3.0, 3.3)	3.2 (3.1, 3.3)	5.3 (4.7, 6.0)
**median**	1.0 (1.0, 1.0)	2.0 (1.0, 2.0)	3.0 (2.0, 3.0)
**std**	5.9 (5.5, 6.4)	6.0 (5.6, 6.5)	7.4 (6.0, 9.3)
**min**	1.0 (1.0, 1.0)	1.0 (1.0, 1.0)	1.0 (1.0, 1.0)
**max**	110 (87, 110)	110 (88, 110)	72 (35, 72)
**5%**	1.0 (1.0, 1.0)	1.0 (1.0, 1.0)	1.0 (1.0, 1.0)
**95%**	10 (10, 10)	11 (10, 11)	19 (15, 25)

S3.7. Regular vs. Irregular Conditions

In this section, we compare the descriptive statistics of all regularly operating facilities excluding Kiyanly LNG (results provided in the main manuscript), all regularly operating facilities including Kiyanly LNG, Kiyanly LNG only, and facilities with an interrupted status

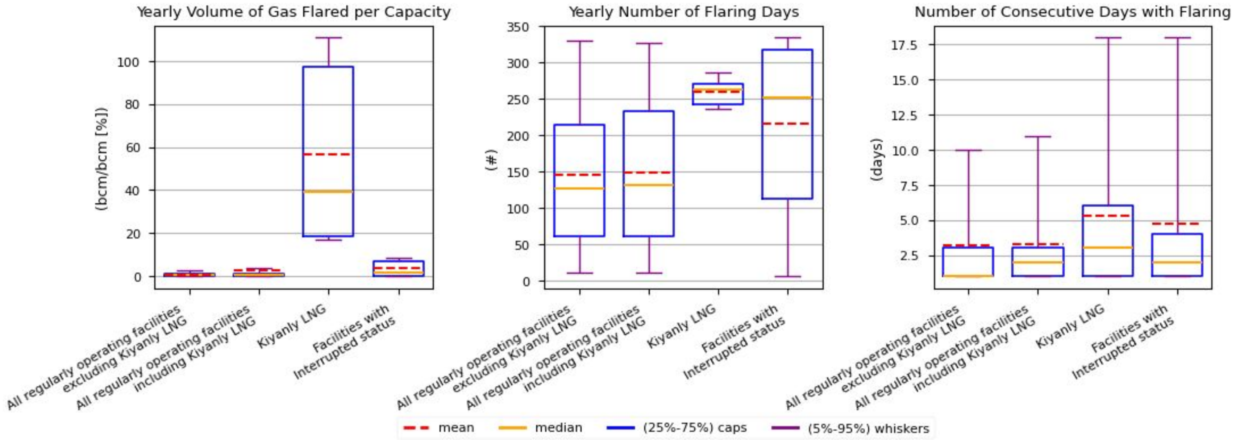

Figure S17. Analysis of yearly numbers of flaring days, number of consecutive days with flaring, and volumes of gas flared per capacity for facilities with a continuous status excluding Kiyanly LNG, facilities with continuous status including Kiyanly LNG, Kiyanly LNG only, and facilities with an interrupted status.

Table S26. Descriptive statistics of yearly volume of gas flared per capacity (bcm/bcm [%]) for all regularly operating facilities excluding Kiyanly LNG, for all regularly operating facilities including Kiyanly LNG, for Kiyanly LNG and for all facilities with an interrupted status. The numbers in brackets correspond to the 95% confidence interval (i.e, 2.5% - 97.5%).

	**All regularly operating facilities excluding Kiyanly LNG**	**All regularly operating facilities including Kiyanly LNG**	**Kiyanly LNG**	**All facilities with an interrupted status**
**mean**	0.84 (0.72, 1.02)	2.7 (1.6, 4.6)	56 (32, 81)	3.6 (2.4, 4.7)
**median**	0.47 (0.41, 0.55)	0.50 (0.42, 0.57)	39 (17, 96)	1.7 (0.39, 5.0)
**std**	1.2 (0.85, 1.7)	13 (6.8, 18)	41 (33, 47)	3.5 (2.9, 4.0)
**min**	0.0038 (0.0038, 0.0038)	0.0038 (0.0038, 0.0038)	15 (15, 15)	0.016 (0.016, 0.016)
**max**	13 (3.9, 13)	116 (19, 116)	116 (51, 116)	11 (7.8, 11)
**5%**	0.059 (0.041, 0.088)	0.06 (0.046, 0.092)	16 (15, 17)	0.034 (0.016, 0.14)
**95%**	2.7 (2.3, 3.5)	4.1 (2.7, 15)	113 (73, 116)	9.1 (7.4, 11)

Table S27. Descriptive statistics of number of flaring days per year (#) for all regularly operating facilities excluding Kiyanly LNG, for all regularly operating facilities including Kiyanly LNG, for Kiyanly LNG and for all facilities with an interrupted status. The numbers in brackets correspond to the 95% confidence interval (i.e, 2.5% - 97.5%).

	**All regularly operating facilities excluding Kiyanly LNG**	**All regularly operating facilities including Kiyanly LNG**	**Kiyanly LNG**	**All facilities with an interrupted status**
**mean**	145 (133, 155)	149 (137, 159)	259 (246, 270)	215 (170, 255)
**median**	127 (112, 138)	131 (120, 145)	262 (235, 266)	251 (119, 303)
**std**	101 (95, 106)	101 (96, 107)	20 (14, 25)	115 (93, 133)
**min**	3.0 (3.0, 3.0)	3.0 (3.0, 3.0)	227 (227, 227)	1.0 (1.0, 1.0)
**max**	353 (341, 353)	353 (343, 353)	287 (264, 287)	341 (323, 341)
**5%**	9.9 (7.0, 16)	10 (7.0, 15)	23 (227, 238)	5.5 (1.0, 51)
**95%**	330 (322, 335)	329 (320, 335)	287 (266, 287)	335 (323, 341)

Table S28. Descriptive statistics of number of consecutive days with flaring (days) for all regularly operating facilities excluding Kiyanly LNG, for all regularly operating facilities including Kiyanly LNG, for Kiyanly LNG and for all facilities with an interrupted status. The numbers in brackets correspond to the 95% confidence interval (i.e, 2.5% - 97.5%).

	**All regularly operating facilities excluding Kiyanly LNG**	**All regularly operating facilities including Kiyanly LNG**	**Kiyanly LNG**	**All facilities with an interrupted status**
**mean**	3.2 (3.1, 3.3)	3.3 (3.1, 3.4)	5.3 (4.7, 6.0)	4.8 (4.3, 5.2)
**median**	1.0 (1.0, 1.0)	2.0 (1.0, 2.0)	3.0 (2.0, 3.0)	2.0 (2.0, 2.0)
**std**	6.0 (5.5, 6.4)	6.1 (5.7, 6.5)	7.4 (6.0, 9.3)	9.1 (7.4, 11)
**min**	1.0 (1.0, 1.0)	1.0 (1.0, 1.0)	1.0 (1.0, 1.0)	1.0 (1.0, 1.0)
**max**	110 (89, 110)	110 (94, 110)	72 (35, 72)	122 (62, 122)
**5%**	1.0 (1.0, 1.0)	1.0 (1.0, 1.0)	1.0 (1.0, 1.0)	1.0 (1.0, 1.0)
**95%**	10 (10, 10)	11 (10, 11)	19 (15, 25)	18 (15, 20)

S3.8. Case 4: Offshore vs. Onshore

In this section, we compare the descriptive statistics of all onshore facilities excluding Kiyanly LNG (results provided in the main manuscript), all onshore facilities including Kiyanly LNG, Kiyanly LNG only, and offshore facilities.

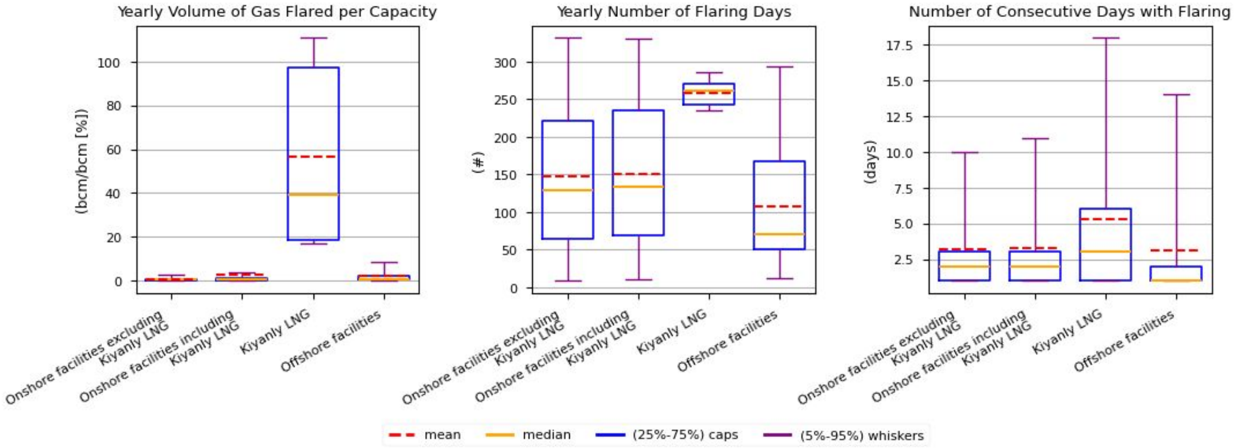

Figure S18. Analysis of yearly numbers of flaring days, number of consecutive days with flaring, and volumes of gas flared per capacity for onshore facilities, onshore facilities including Kiyanly LNG, only Kiyanly LNG, and offshore facilities.

Table S29. Descriptive statistics of yearly volume of gas flared per capacity (bcm/bcm [%]) for all onshore facilities excluding Kiyanly LNG, for all onshore facilities including Kiyanly LNG, for Kiyanly LNG and for all offshore facilities. The numbers in brackets correspond to the 95% confidence interval (i.e, 2.5% - 97.5%).

	**All onshore facilities excluding Kiyanly LNG**	**All onshore facilities including Kiyanly LNG**	**Kiyanly LNG**	**All offshore facilities**
**mean**	0.75 (0.65, 0.86)	2.8 (1.5, 4.7)	56 (32, 81)	2.0 (0.94, 3.7)
**median**	0.47 (0.39, 0.55)	0.50 (0.41, 0.57)	39 (17, 96)	0.56 (0.35, 1.7)
**std**	0.89 (0.72, 1.1)	13 (6.5, 18.5)	41 (33, 47)	3.2 (1.1, 4.8)
**min**	0.0038 (0.0038, 0.0038)	0.0038 (0.0038, 0.0038)	15 (15, 15)	0.082 (0.082, 0.082)
**max**	6.9 (3.7, 6.9)	116 (27, 116)	116 (51, 116)	13 (2.3, 13)
**5%**	0.058 (0.04, 0.089)	0.058 (0.041, 0.095)	16 (15, 17)	0.082 (0.082, 0.092)
**95%**	2.6 (2.1, 2.9)	3.9 (2.7, 17)	113 (73, 116)	8.7 (2.3, 13)

Table S30. Descriptive statistics of number of flaring days per year (#) for all onshore facilities excluding Kiyanly LNG, for all onshore facilities including Kiyanly LNG, for Kiyanly LNG and for all offshore facilities. The numbers in brackets correspond to the 95% confidence interval (i.e, 2.5% - 97.5%).

	All onshore facilities excluding Kiyanly LNG	All onshore facilities including Kiyanly LNG	Kiyanly LNG	All offshore facilities
**mean**	147 (136, 159)	151 (140, 163)	259 (246, 270)	107 (71, 147)
**median**	130 (119, 145)	134 (122, 149)	262 (235, 266)	71 (50, 131)
**std**	101 (95, 107)	102 (96, 107)	20 (14, 25)	89 (64, 111)
**min**	3.0 (3.0, 3.0)	3.0 (3.0, 3.0)	227 (227, 227)	10 (10, 10)
**max**	353 (340, 353)	353 (341, 353)	287 (264, 287)	296 (176, 296)
**5%**	9.0 (6.9, 16)	9.4 (7.0, 17)	23 (227, 238)	12 (10, 14)
**95%**	332 (322, 337)	331 (322, 335)	287 (266, 287)	293 (167, 296)

Table S31. Descriptive statistics of number of consecutive days with flaring (days) for all onshore facilities excluding Kiyanly LNG, for all onshore facilities including Kiyanly LNG, for Kiyanly LNG and for all offshore facilities. The numbers in brackets correspond to the 95% confidence interval (i.e, 2.5% - 97.5%).

	All onshore facilities excluding Kiyanly LNG	All onshore facilities including Kiyanly LNG	Kiyanly LNG	All offshore facilities
**mean**	3.2 (3.1, 3.3)	3.3 (3.2, 3.4)	5.3 (4.7, 6.0)	3.2 (2.7, 3.7)
**median**	2.0 (1.0, 2.0)	2.0 (1.0, 1.0)	3.0 (2.0, 3.0)	1.0 (1.0, 1.0)
**std**	6.0 (5.5, 6.5)	6.1 (5.6, 6.5)	7.4 (6.0, 9.3)	6.3 (4.5, 8.7)
**min**	1.0 (1.0, 1.0)	1.0 (1.0, 1.0)	1.0 (1.0, 1.0)	1.0 (1.0, 1.0)
**max**	110 (88, 110)	110 (87, 110)	72 (35, 72)	94 (32, 94)
**5%**	1.0 (1.0, 1.0)	1.0 (1.0, 1.0)	1.0 (1.0, 1.0)	1.0 (1.0, 1.0)
**95%**	10 (10, 10)	11 (10, 11)	19 (15, 25)	14 (8.0, 16)

S3.9. Probabilities of Flaring Events and Associated Volumes of Gas Flared per Life Stage

Table S32 Flaring events and associated volumes of gas flared per life-cycle stage (i.e., start-up and regular operating years), estimated independently from each other, for various probabilities (1-exceedance probability). The numbers in brackets represent the 95% confidence interval (2.5% - 97.5%)

Operation Type	Probability [%]	Yearly volumes of gas flared per capacity (bcm/bcm [%])	Number of flares detected per year (#)	Number of consecutive days with flaring (days)
**Regular Operation <u>excluding</u> Kiyanly LNG**	90	0.0983 (0.0747, 0.128)	26.9 (19.2, 36.4)	0.92 (0.914, 0.926)
	80	0.176 (0.149, 0.208)	59.9 (50.3, 70.7)	1.01 (1, 1.01)
	70	0.255 (0.215, 0.297)	86.2 (74.9, 99.5)	1.12 (1.11, 1.13)
	60	0.345 (0.287, 0.397)	110 (97.9, 126)	1.27 (1.25, 1.29)
	50	0.452 (0.378, 0.521)	135 (121, 151)	1.49 (1.46, 1.51)
	40	0.588 (0.501, 0.677)	161 (146, 178)	1.81 (1.77, 1.84)
	30	0.774 (0.665, 0.894)	190 (175, 208)	2.33 (2.28, 2.39)
	20	1.06 (0.916, 1.23)	228 (212, 246)	3.32 (3.23, 3.4)
	10	1.64 (1.4, 1.91)	285 (269, 303)	5.83 (5.63, 6.04)
**Regular Operation <u>including</u> Kiyanly LNG**	90	0.166 (0.138, 0.204)	28.1 (20.2, 36.7)	0.916 (0.909, 0.921)
	80	0.181 (0.152, 0.22)	63.4 (53.6, 74.6)	1.01 (1, 1.01)
	70	0.206 (0.175, 0.244)	91 (79.6, 104)	1.13 (1.12, 1.14)
	60	0.249 (0.211, 0.291)	116 (103, 131)	1.29 (1.27, 1.31)
	50	0.324 (0.265, 0.381)	141 (128, 157)	1.52 (1.49, 1.54)
	40	0.459 (0.367, 0.553)	167 (153, 184)	1.86 (1.82, 1.9)
	30	0.728 (0.572, 0.93)	197 (182, 214)	2.41 (2.36, 2.47)
	20	1.36 (1.05, 1.96)	234 (219, 250)	3.44 (3.35, 3.53)
	10	3.55 (2.49, 5.97)	290 (275, 305)	6.05 (5.86, 6.26)

S4. Discussion

S4.1. Start-Up Conditions in Environmental Impact Assessments

The environmental impact assessment (EIA) for the Sabine Pass (U.S.) facility (opened in 2016) states that the start-up steps would extend over one to two months and show higher flaring emissions than under regular operating conditions[1]. Proponents for the Corpus Christi (U.S.) facility (2018) simply indicated that the start-up process of the terminal "*would result in larger emissions than under normal operating conditions and would last for several months*"[2]. The QC LNG (Australia, 2015) EIA states that start-up could lead to "*periodic flaring for up to five days*", but we could not find a mention of duration of this start-up phase[3]. In Kitimat, BC,

Canada, the first Canadian LNG export facility (LNG Canada) started operating early 2024, with the first exports of LNG to be scheduled mid-2025[4], corresponding to almost 2 years of planned start-up operations. In a recent update, the proponents provided estimates of volumes of gas flared during the testing of the flaring system of the facility, which should occur over a 10-day period (i.e., "*approximately 150,000 cubic metres per hour, with a rate of up to approximately 100 tons/day*")[5]. We could not find this information in LNG Canada's EIA, suggesting that it is unlikely an air quality modeling under those conditions was conducted[6].

Nevertheless, many facilities only briefly mention start-up conditions in their air quality impact assessments. Freeport LNG's proponents (U.S., 2019) analyzed flaring under start-up conditions solely in their noise impact assessment, and mentioned start-up only when describing reporting regulations for excessive emissions[7]. In the Gladstone LNG (Australia, 2015) and AP LNG (2016) air quality impact statements, start-up was solely associated with scheduled shutdown and maintenance of the facilities and did not refer to the initial commissioning phase, which was ultimately not considered[8,9]. In the case of PN LNG (Papua New Guinea, 2013), proponents did not mention start-up conditions at all[10]. This pattern is repeated in the EIA for British Columbia's Woodfibre LNG facility under construction[11].

S4.2. Maintenance Flaring in Environmental Impact Assessments

The EIA report for Corpus Christi (U.S.) indicates the amount of gas that would need to be purged during a maintenance event[61]. Similarly, Freeport LNG (U.S.) has a permit that stipulates how much gas could be flared during maintenance start-up and shutdown on a rolling 12-month period[63], but this does not provide measurements on volumes of gas expected to be or actually flared during upset/maintenance conditions. Scenarios with excess flaring provided for Gladstone LNG (Australia) and modeled for air quality impact assessment only included scheduled maintenance indicated to occur every three years and lasting three hours[64].

References

1 Sabine Pass Liquefaction LLC and Sabine Pass LNG, *Environmental Assessment for the Sabine Pass Liquefaction Project*, 2011.

2 C. C. L. L. and C. C. C. P. L.P., *Corpus Christi LNG Project - Final Environmental Impact Assessment*, 2014.

3 QGC LIMITED, *QUEENSLAND CURTIS LNG Volume 5: Chapter 12 Air Quality*, 2009.

4 M. Čavčić, *Offshore Energy*, 2024.

5 LNG Canada, Community Notification: Increased Flaring Activity, https://www.lngcanada.ca/news/community-notification-increased-flaring-activity/.

6 BC Environmental Assessment Office (EAO), LNG Canada Environmental Assessment, https://projects.eao.gov.bc.ca/p/588511d0aaecd9001b826192/project-details.

7 F. L. 3 L. Freeport LNG Development L.P, FLNG Liquefaction LLC, FLNG Liquefaction 2 LLC, *Freeport LNG Liquefaction Project Phase II Modification Project Final Environmental Impact Assessment*, 2014.

8 Santos Ltd, *GLNG - Environmental Impact Statement - Air Quality*, 2009.

9 Australia Pacific LNG, *Australia Pacific LNG Project EIS Volume 4: LNG Facility Chapter 13: Air Quality*, 2010.

10 Holmes Air Sciences, *AIR QUALITY ASSESSMENT: PAPUA NEW GUINEA LIQUEFIED NATURAL GAS PROJECT (LNG FACILITIES)*, 2009.

11 BC Environmental Assessment Office (EAO), Woodfibre LNG Environmental Assessment, https://projects.eao.gov.bc.ca/p/588511e1aaecd9001b8272e7/project-details.
